# Enhanced Bifunctional Electrocatalysis for Zinc‐Air Battery Using Porous Conductive Substrate with Abundant Anchoring Sites

**DOI:** 10.1002/advs.202506172

**Published:** 2025-07-28

**Authors:** Jongkyoung Kim, Je Min Yu, Jun‐Yong Choi, Seong‐Hun Lee, Han Uk Lee, Dongrak Oh, Hyunju Go, Wonsik Jang, Seunghyun Lee, Jaewon Cho, Sung Beom Cho, Tae Joo Shin, Hyunjoo Lee, Sang‐Goo Lee, Ji‐Wook Jang, Seungho Cho, Wook Jo

**Affiliations:** ^1^ Department of Materials Science and Engineering Ulsan National Institute of Science and Technology (UNIST) Ulsan 44919 Republic of Korea; ^2^ School of Energy and Chemical Engineering Ulsan National Institute of Science and Technology (UNIST) Ulsan 44919 Republic of Korea; ^3^ Graduate School of Semiconductor Materials and Devices Engineering Ulsan National Institute of Science and Technology (UNIST) Ulsan 44919 Republic of Korea; ^4^ Department of Energy Systems Research Ajou University Suwon Gyeonggi‐do 16499 Republic of Korea; ^5^ Center for Future Semiconductor Technology (FUST) Ulsan National Institute of Science and Technology (UNIST) Ulsan 44919 Republic of Korea; ^6^ iBUle Photonics, Inc. Incheon 21999 Republic of Korea

**Keywords:** bifunctional electrocatalysts, heterostructures, oxygen evolution reaction, oxygen reduction reaction, zinc‐air batteries

## Abstract

Efficient and robust bifunctional electrocatalysts for oxygen evolution reaction (OER) and oxygen reduction reaction (ORR) are critical for high‐performance zinc‐air batteries (ZABs). However, balancing OER and ORR activity in a single catalyst remains challenging due to the different mechanisms during charging and discharging. Here, a scalable strategy is presented for enhancing both reactions by integrating two‐dimensional OER‐ and ORR‐active components onto a carbon‐based conductive substrate with abundant anchoring sites, via high‐shear exfoliation. The heterostructure catalyst demonstrates exceptional bifunctionality, achieving an extremely low overpotential difference of 0.63 V. First‐principles calculations confirm a strong chemical compatibility between the active components and substrate. In scaled‐up ZAB applications, the catalyst delivers a high peak power density of 1569 mW cm^−2^, and an outstanding cycling stability over 300 h (1800 cycles). This work highlights a versatile approach for designing multifunctional electrocatalysts, advancing scalable energy conversion and storage technologies.

## Graphical abstract

A scalable nanocomposite synthesis is reported for a high‐performance bifunctional electrocatalyst by combining exfoliated layered materials—each active for OER and ORR—with a porous conductive substrate via high‐shear exfoliation. The electrocatalyst exhibits the lowest potential gap among reported transition metal‐based bifunctional catalysts. In a scaled‐up zinc‐air battery, the system demonstrates outstanding stability over 300 h.

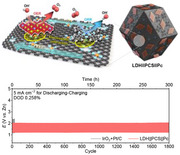

## 1 Introduction

The increasing global demand for sustainable energy solutions, driven by the ongoing energy crisis and environmental pollution, has intensified research on advanced energy storage technologies. Rechargeable zinc‐air batteries (ZABs) have drawn significant attention as a promising candidate due to their cost‐effectiveness, high theoretical energy density, and inherent safety.^[^ ^1^ ^]^ However, despite their potential, ZABs face critical challenges stemming from the sluggish kinetics of oxygen evolution reaction (OER) and oxygen reduction reaction (ORR), which limit their overall efficiency and power output. While noble metal‐based electrocatalysts exhibit excellent catalytic activity for OER and ORR, their high cost and limited bifunctionality as a single element hinder their practical application in ZABs.^[^ ^2^ ^]^ A practical strategy for enhancing bifunctional catalytic performance involves combining distinct active materials for OER and ORR, forming a composite system.^[^ ^3^ ^]^ Nevertheless, integrating heterogeneous materials often encounters challenges, such as low binding affinity among components, leading to phase separation through self‐aggregation. This separation disrupts efficient electron transfer between materials and significantly degrades electrochemical performance during prolonged battery cycling.^[^ ^4^ ^]^

Transition metal‐based layered double hydroxides (LDHs) are two‐dimensional (2D) materials composed of positively charged nanosheets, known for their high OER performance due to uniform distribution of metal cations.^[^ ^5^ ^]^ Similarly, iron‐based phthalocyanine (FePc) is a promising 2D ORR catalyst with Fe single atoms coordinated to nitrogen (N) atoms providing exceptional activity.^[^ ^6^ ^]^ Despite their intrinsic catalytic efficiency, the practical utilization of these 2D materials is hindered by their bulk 3D layered structures with weak out‐of‐plane interactions, leading to self‐aggregation in the absence of a suitable substrate.^[^ ^7^ ^]^ Efficient electron transfer during catalysis further necessitates a highly conductive substrate. However, conventional conductive substrates such as carbon black, carbon nanotubes, and graphene often lack sufficient binding sites to anchor diverse 2D materials effectively.^[^ ^8^ ^]^ Addressing these challenges requires the development of substrates that are not only highly conductive but also rich in anchoring sites for stabilizing 2D nanosheets and, thus, fully exploit their catalytic potential.

Herein, we present a rational synthetic strategy, supported by theoretical calculations, to develop a bifunctional electrocatalyst by anchoring OER‐active and ORR‐active 2D nanosheets onto a conductive substrate via a high‐shear exfoliation (HSE) method. CoNiFe‐based LDH nanosheets and FePc nanosheets were utilized as OER and ORR catalysts, respectively. A porous conductive substrate (denoted as PCS) was synthesized through the pyrolysis of a zeolitic imidazolate framework (ZIF), yielding a highly conductive material with well‐dispersed cobalt (Co) and N species at the nanoscale. This hierarchical structure provides abundant anchoring sites with a high surface area, enabling robust interfacial interactions between the active components and substrate. Therefore, a nanocomposite comprising exfoliated CoNiFe LDH and FePc nanosheets anchored onto the conductive PCS (denoted as LDH||PCS||Pc) was successfully fabricated. Leveraging the synergistic effects between the active 2D nanosheets and the conductive substrate, the nanocomposite exhibited exceptional bifunctional catalytic performance (Δ*E*  =  0.63 V), surpassing both commercial IrO_2_ (for OER) and Pt/C (for ORR) catalysts and most of the previously reported bifunctional catalysts. Furthermore, the performance of this advanced bifunctional electrocatalyst was validated through its integration into a zinc‐air battery (ZAB) system, demonstrating significant advancements over conventional designs. The LDH||PCS||Pc‐based ZAB exhibited superior electrochemical performance, including a higher open‐circuit potential (1.57 V) and a similar peak power density (172 mW cm^−2^) compared with the IrO_2_+Pt/C‐based ZAB. Remarkably, when scaled up with an optimized system architecture and flowing‐electrolyte design, the LDH||PCS||Pc‐based large‐scale ZAB achieved a 9.12‐fold improvement in peak power density (1569 mW cm^−2^) and exceptional stability over 300 h for a continuous operation. This study highlights a facile and scalable approach for fabricating high‐performance electrocatalysts through the integration of active materials and conductive substrates, offering significant potential for cost‐effective applications across various technological fields.

## 2 Results and Discussion

### 2.1 Synergistic Integration via High‐Shear Exfoliation for Bifunctional Electrocatalyst

**Figure 1:**
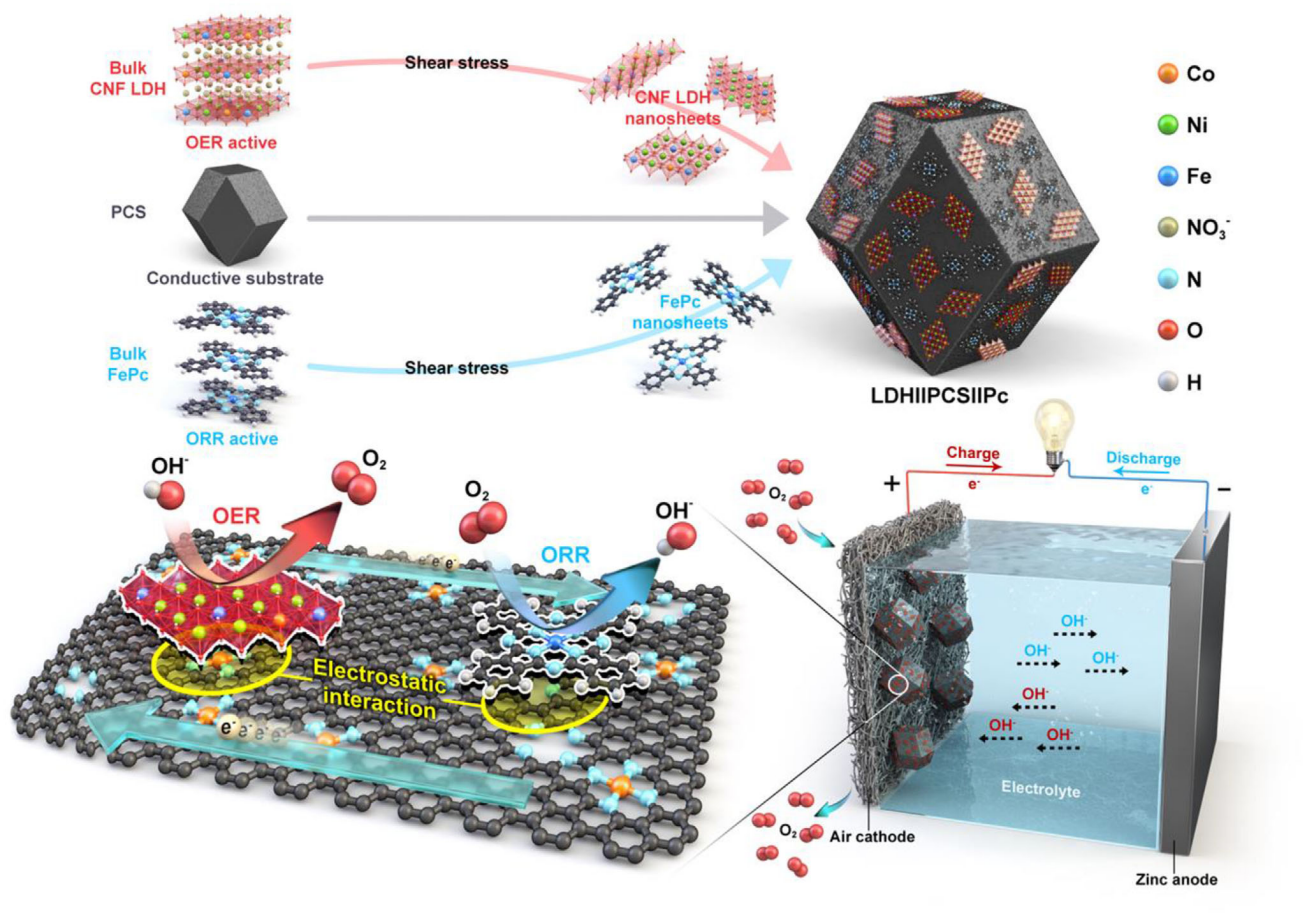
Schematic illustration of the synergistic OER/ORR bifunctional activity of the LDH||PCS||Pc electrocatalyst.

We propose the following synthetic strategies for the development of a superior bifunctional OER/ORR catalyst. 1) We selected CoNiFe‐based LDH (denoted as CNF LDH) and FePc as active layered materials owing to their high catalytic performance in OER and ORR, respectively. 2) Additionally, PCS was chosen to prevent nanosheet self‐aggregation and promote efficient electron transfer (detailed in Note S1 and Figure S1, Supporting Information).^[^ ^9^ ^]^ 3) As illustrated in **Figure** **1**, exfoliated CNF LDH and FePc nanosheets were anchored onto PCS to achieve their uniform dispersion (detailed in the Experimental Section). The LDH||PCS||Pc nanocomposite was prepared using a high‐shear homogenizer, which enabled simultaneous exfoliation and material integration, facilitating scalable production.

In this process, bulk CNF LDH, PCS, and bulk FePc were immersed in an aqueous solution. Upon initiation of high‐speed rotation, the solution was drawn into the high‐shear region between the stator and rotor. The resulting centrifugal forces generated lateral and longitudinal shear forces, facilitating the exfoliation of CNF LDH and FePc by disrupting the electrostatic interactions within their bulk layered structures (Note S2 and Figure S2, Supporting Information).^[^ ^10^ ^]^ Simultaneously, the PCS electrostatically interacted with the exfoliated nanosheets through its abundant anchoring sites, preventing self‐aggregation. This approach resulted in robust interfacial interactions between PCS and the nanosheets (CNF LDH and FePc), forming a stable heterogeneous architecture, underscoring the potential of this method for scalable and efficient electrocatalyst fabrication.

### 2.2 DFT Study on Chemical Adhesion between PCS and Exfoliated Nanosheets

**Figure 2:**
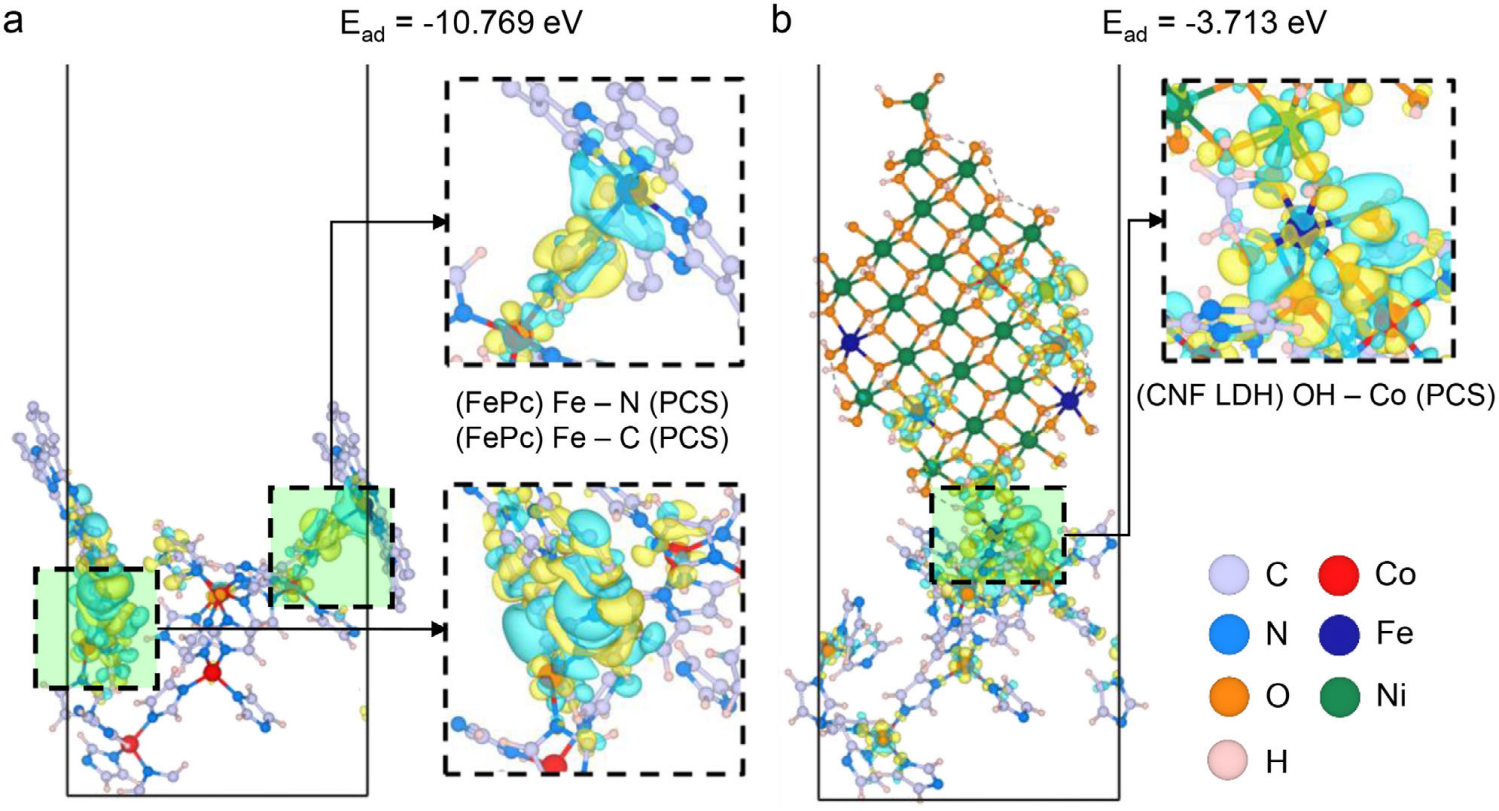
DFT calculation results of interfaces. Adsorption energy and charge density difference between a) PCS‐FePc, and b) PCS‐CNF LDH.

To validate the chemical adsorption behavior of FePc and CNF LDH nanosheets on PCS, density functional theory (DFT) calculations were conducted (**Figure** **2**). Atomic models of PCS, FePc, and CNF LDH were designed to closely replicate experimental conditions. The PCS model was derived from the pristine ZIF‐8 atomic structure by randomly mixing Co and Zn atoms at a 1:1 ratio, followed by selective removal of Zn atoms and associated functional groups (C, N, and H). The FePc model consisted of a single molecular layer extracted from a bulk FePc cluster, while the CNF LDH model featured a monolayer with a Co:Ni:Fe atomic ratio of 1:9:3 (Figure S3a,b, Supporting Information). In addition, radial distribution function (RDF) of each model showed the specific atomic pairs and their interatomic distances (Figure S3c, Supporting Information).

The adsorption energies for PCS‐FePc (Figure 2a) and PCS‐CNF LDH (Figure 2b) were calculated using the formula, *E_ad_* = *E_total_*  − *E_PCS_* − *E_Pc_* (*or* *E_LDH_*). The adsorption energy of PCS‐FePc is −10.769 eV, while that of PCS‐CNF LDH is −3.713 eV. For FePc nanosheets, Fe atoms formed bonds with C or N atoms in PCS, while OH groups from CNF LDH nanosheets bonded with Co atoms in PCS. Both PCS‐FePc and PCS‐CNF LDH exhibited negative adsorption energies, indicating thermodynamically favorable adhesion. Moreover, significant charge transfers were observed at the adsorption sites, suggesting strong interaction between PCS and the active materials (exfoliated FePc and CNF LDH nanosheets).

### 2.3 Structural Characterizations of LDH||PCS||Pc

**Figure 3:**
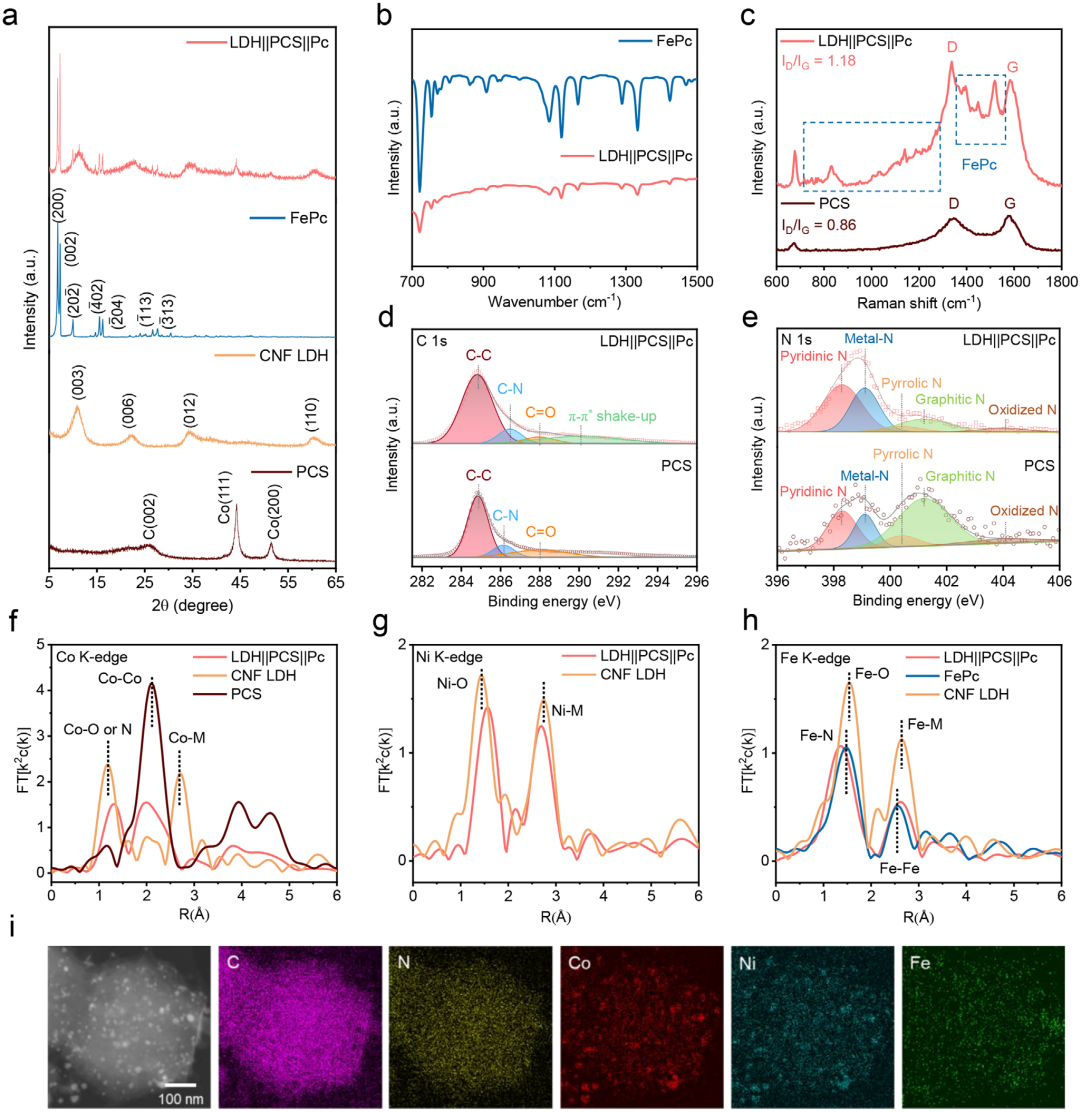
Characterization of LDH||PCS||Pc, FePc, CNF LDH and PCS. a) X‐ray diffraction patterns of LDH||PCS||Pc, FePc, CNF LDH and PCS. b) FT‐IR spectra of LDH||PCS||Pc and FePc. c) Raman spectra of LDH||PCS||Pc and PCS. X‐ray photoelectron spectroscopy (XPS) spectra of LDH||PCS||Pc and PCS for d) C 1s and e) N 1s. f) FT k^2^‐weighted R‐space Co K‐edge extended X‐ray absorption fine structure spectra of LDH||PCS||Pc, CNF LDH and PCS. g) FT k^2^‐weighted R‐space Ni K‐edge extended X‐ray absorption fine structure spectra of LDH||PCS||Pc, CNF LDH. h) FT k^2^‐weighted R‐space Fe K‐edge extended X‐ray absorption fine structure spectra of LDH||PCS||Pc, CNF LDH, and FePc. i) TEM image and corresponding elemental maps of LDH||PCS||Pc.

The crystalline phases of LDH||PCS||Pc, FePc, CNF LDH, and PCS were characterized by X‐ray diffraction (XRD) patterns (**Figure** **3**a). FePc, CNF LDH, and PCS displayed characteristic XRD patterns consistent with their respective crystal structure.^[^ ^7^, ^11^ ^]^ PCS showed three distinct peaks approximately at 26°, 44°, and 51°, corresponding to the (002) plane of graphitic carbon and the (111) and (200) plane of the metallic Co, respectively.^[^ ^11a^ ^]^ Atomic force microscopy (AFM) imaging and corresponding height profile of CNF LDH nanosheets revealed a height of ≈0.9 nm, closely matching the theoretical thickness of an individual LDH nanosheet (≈0.8 nm), accompanied by a pronounced Tyndall effect (Figure S4, Supporting Information).^[^ ^12^ ^]^ Similarly, the AFM image and height profile of FePc nanosheets (Figure S5, Supporting Information) exhibited a thickness of ≈1.8 nm, corresponding to five layers, along with a distinct Tyndall effect.^[^ ^13^ ^]^ These results confirm the effectiveness of the HSE method in producing well‐exfoliated 2D materials.

LDH||PCS||Pc nanocomposite retained all the characteristic XRD peaks of FePc, CNF LDH, and PCS without any alterations in their crystal structures, indicating that the HSE method had minimal impact on crystallinity. To further confirm the successful incorporation of FePc into the nanocomposite, Fourier transform infrared (FT‐IR) spectroscopy was performed (Figure 3b). The FT‐IR spectrum of LDH||PCS||Pc exhibited distinct characteristic peaks corresponding to FePc, verifying its presence within the composite structure.^[^ ^14^ ^]^ Raman spectroscopy was carried out to assess the degree of the disorder in the carbon materials (Figure 3c). The disorder was assessed using two characteristic peaks: the D‐band (≈1342 cm^−1^), indicative of disorder, and the G‐band (≈1575 cm^−1^), associated with graphitic carbon.^[^ ^15^ ^]^ The LDH||PCS||Pc nanocomposite displayed a higher D/G intensity ratio (I_D_/I_G_) of ≈1.18 compared to PCS alone (≈0.86), suggesting an increased degree of disorder. This increase reflects strong interactions between PCS and the active materials (CNF LDH and FePc) and is attributed to nitrogen atoms located on defective edges derived from in‐plane pores in Co‐involved samples.^[^ ^8c^ ^]^ Furthermore, the Raman spectrum of LDH||PCS||Pc showed prominent characteristic peaks of FePc, confirming the successful coupling of PCS with the active materials (Figure S6, Supporting Information). In addition, nitrogen adsorption–desorption measurements were conducted to assess the surface area and pore characteristics of each sample using the Brunauer–Emmett–Teller (BET) and Barrett–Joyner–Halenda (BJH) methods (Figure S7 and Table S1, Supporting Information). As shown in Figure S7a–c (Supporting Information), CNF LDH and PCS exhibited mesoporous structures, whereas FePc displayed a non‐porous profile. Among the samples, PCS showed the highest surface area and pore volume, confirming its potential as an effective porous substrate. After the HSE process, CNF LDH and FePc demonstrated substantial increases in specific surface area, attributed to reduced stacking order and the formation of loosely restacked assemblies—indicative of successful exfoliation (Figure S7d–f, Supporting Information).^[^ ^5^, ^16^ ^]^ In contrast, PCS exhibited minimal change in surface area due to its intrinsic 3D structure. Consequently, the composite LDH||PCS||Pc retained a mesopore‐dominated character (Figure S7g, Supporting Information).

X‐ray photoelectron spectroscopy (XPS) was conducted to analyze the electronic states of C and N within the carbon matrix. In LDH||PCS||Pc, a positive shift in the C‐N binding energy was observed, indicating electronic coupling between PCS and FePc (Figure 3d). Additionally, the nanocomposite exhibited a distinct π‐π^*^ shake‐up peak ≈288 eV, associated with the aromatic C structure of FePc.^[^ ^17^ ^]^ The N 1s spectra for all samples were deconvoluted into five main peaks centered at ≈398.5, 399.5, 400.8, 401.2, and 403.5 eV, corresponding to pyridinic N, metal‐N, pyrrolic N, graphitic N, and oxidized N, respectively (Figure 3e).^[^ ^18^ ^]^ Notably, LDH||PCS||Pc showed an increased intensity of pyridinic‐N, which potentially contributes to optimizing the adsorption of reaction intermediates and enhancing the reversible OER and ORR processes.^[^ ^18b^ ^]^

To elucidate the local structural environment of transition metals, extended X‐ray absorption fine structure (EXAFS) analysis was conducted at the K‐edge energy of Co, Ni, and Fe. For CNF LDH, the EXAFS spectrum of Co exhibited prominent peaks at 1.18 and 2.71 Å, corresponding to Co─O and Co─M (M = Ni, Co, Fe) bonds, respectively (Figure 3f).^[^ ^19^ ^]^ In contrast, the Co spectrum for PCS showed a dominant peak at 2.11 Å and additional peaks at 3.25, 3.93, and 4.59 Å, representing Co─Co bonds with distinct bonding distances, consistent with metallic Co. However, in the LDH||PCS||Pc nanocomposite, the numerous peaks from PCS were significantly diminished, reflecting interactions between PCS and active components during the HSE process.^[^ ^20^ ^]^ Concurrently, the Co─N or ─O peak intensity increased, indicating the formation of new interactions between Co sites of PCS and CNF LDH or FePc. A similar trend was observed for Ni. In CNF LDH, Ni exhibited peaks at 1.44 and 2.75 Å, corresponding to Ni─O and Ni─M bonds, respectively, which shifted to 1.57 and 2.69 Å in LDH||PCS||Pc (Figure 3g). For Fe, the EXAFS spectrum of CNF LDH displayed peaks at 1.55 and 2.64 Å, attributed to Fe─O and Fe─M bonds, respectively (Figure 3h). FePc exhibited peaks at 1.47 and 2.55 Å, corresponding to Fe─N and Fe─Fe bonds, respectively. In the LDH||PCS||Pc composite, the peak at 1.35 Å increased, indicating a shortening of Fe─N or O bonds, while the Fe─M bond lengthened due to steric effects. These peak trends were consistent with dominant peaks of RDF from our DFT model, supporting the reliability of the EXAFS data (Figure S3c, Supporting Information). These results collectively demonstrate a strong interaction between PCS and the active components (CNF LDH and FePc), as evidenced by the shifts in bond distances and changes in the EXAFS spectra, thereby indicating enhanced structural and electronic coupling within the nanocomposite.

Transmission electron microscopy (TEM) images and the corresponding elemental maps of PCS revealed uniformly distributed Co nanoparticles on the polyhedral carbon matrix, with a homogeneous distribution of C and N (Figure S8, Supporting Information). As shown in Figure 3i, elemental mappings confirmed the uniform distribution of all elements on the substrate, verifying the successful anchoring of both the CNF LDH and FePc nanosheets on PCS. Additionally, the overlapping positions of Co and Ni further corroborate the interaction between CNF LDH and PCS. It is noteworthy that the polyhedral shape of PCS was preserved in the LDH||PCS||Pc nanocomposite (Figure S9, Supporting Information). Metallic Co nanoparticles remained well‐dispersed within the 3D carbon matrix, with an average particle size of ≈9.1 nm (Figure S10, Supporting Information). The atomic ratio of Co, Ni, and Fe in the nanocomposite was quantitatively confirmed through inductively coupled plasma‐optical emission spectroscopy (ICP‐OES) (Table S2, Supporting Information).

### 2.4 Bifunctional Electrochemical Performance of LDH||PCS||Pc

**Figure 4:**
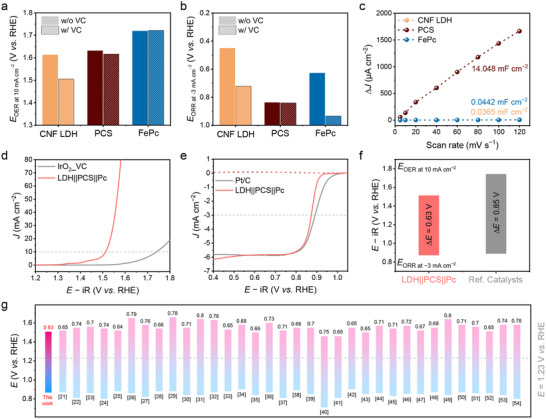
Electrochemical performance of CNF LDH, PCS, FePC, and the LDH||PCS||Pc nanocomposite. Comparison of a) overpotential values at an OER current density of 10 mA cm^−2^ and b) ORR current density of −3 mA cm^−2^ for CNF LDH, PCS, and FePc. c) Double‐layer capacitance (C_dl_) measurement for identifying the electrochemically active surface area (ECSA) of CNF LDH, PCS, and FePc. Linear sweep voltammetry (LSV) curves for determining d) OER and e) ORR electrochemical activity of LDH||PCS||Pc nanocomposite and IrO_2_ with VC (IrO_2__VC) and Pt/C as reference catalysts for each reaction. f) Potential gaps (∆*E*) of LDH||PCS||Pc nanocomposite and reference catalysts. g) Comparison of ∆*E* for state‐of‐the‐art OER/ORR bifunctional catalysts. Details are provided in Table S2 (Supporting Information).

In designing LDH||PCS||Pc as a bifunctional OER/ORR electrocatalyst for zinc‐air battery systems, CNF LDH and FePc were selected as the active sites for the OER and ORR, respectively, while PCS served as a porous support, offering anchoring sites and high conductivity. To evaluate the role of each component, we investigated the electrochemical performance of CNF LDH, PCS, and FePc by loading catalyst ink onto a rotating disk electrode (RDE) for OER and a rotating‐ring disk electrode (RRDE) for ORR in an alkaline electrolyte. The linear sweep voltammetry (LSV) curves for OER are shown in Figure S11 (Supporting Information). The OER potential required to achieve a current density of 10 mA cm^−2^ (*E*_OER_ at 10 mA cm^−2^) for CNF LDH was 1.613 V versus RHE (V_RHE_), lower than that of PCS (1.631 V_RHE_) and FePc (1.719 V_RHE_) (**Figure** **4**a). The high activity of Ni, Co, and Fe‐based LDH catalysts for OER has been reported in previous studies.^[^ ^21^ ^]^ Interestingly, despite its intrinsic catalytic activity, the overpotential of CNF LDH was comparable to that of PCS, which was attributed to the low conductivity of CNF LDH. Thus, we tested the OER performance of the catalysts after incorporating Vulcan carbon black (VC), a commercial conductive substrate, into the catalyst ink. The addition of VC significantly improved the OER performance of CNF LDH, reducing its *E*_OER_ at 10 mA cm^−2^ to 1.505 V_RHE_, whereas the OER performance of PCS and FePc remained largely unchanged.

The ORR performance of CNF LDH, PCS, and FePc was evaluated using RRDE to assess their catalytic activity and electron transfer number during the reaction. ORR can proceed via either a 4‐electron (e^−^) pathway, producing H_2_O or 2‐e^−^ pathway, generating hydrogen peroxide (H_2_O_2_). To determine the electron transfer number, oxygen (O_2_) reduction and H_2_O_2_ oxidation currents were measured using a glassy carbon disk and a platinum (Pt) ring electrode in the RRDE setup. The LSV curves and the potential required to generate ORR current density of −3 mA cm^−2^ (*E*_ORR_ at −3 mA cm^−2^) for all samples are shown in Figure 4b and Figure S12 (Supporting Information). Among the tested materials, PCS exhibited the highest *E*_ORR_ at −3 mA cm^−2^ of 0.838 V_RHE_, and the highest current density of ≈ −5.5 mA cm^−2^. However, its electron transfer number was estimated to be 3.67 at 0.7 V_RHE_ and 3.56 at 0.55 V_RHE_, indicating partial H_2_O_2_ formation, which could promote electrode corrosion in ZAB systems. In contrast, FePc, initially expected to be highly active for ORR, demonstrated relatively low performance. Notably, the addition of VC as a conductive support to the FePc catalyst ink significantly enhanced its activity, resulting in the highest ORR performance among all tested catalysts (Figure 4b; Figure S13, Supporting Information). Furthermore, FePc consistently achieved an electron transfer number close to 4, irrespective of VC addition, confirming its high selectivity and suitability as an ORR catalyst in ZAB systems. These results highlight that CNF LDH and FePc are suitable materials for high‐performance OER and ORR catalysis, respectively in ZAB systems. Moreover, the results underscore the critical role of optimal conductive supports in maximizing the electrochemical performance of each catalyst.

The electrochemically active surface area (ECSA) of the catalysts was assessed by estimating the double‐layer capacitance (C_dl_) to evaluate the extensive active surface area provided by PCS. The ECSA and C_dl_ are calculated as follows:

(1) $$\mathrm{ESCA}=C_{\mathrm{dl}}/C_{s}\;\mathrm{and}\;C_{\mathrm{dl}}=d\left(\Delta J\right)/2dV_{s}$$

where *J* and V_s_ are the current density and scan rate during cyclic voltammetry (CV) measurement in the non‐Faradaic region, respectively, and C_s_ is the specific capacitance of the material, estimating an ideal flat surface. The C_dl_ values were derived from CV curves recorded at varying scan rates (5–120 mV s^−1^). PCS exhibited a significantly higher C_dl_ (14.048 mF cm^−2^), compared CNF LDH (0.0365 mF cm^−2^) and FePc (0.0442 mF cm^−2^) (Figure 4c; Figure S14, Supporting Information). Using a typical C_s_ value of 0.04 mF cm^−2^ in a 1 м alkaline electrolyte,^[^ ^22^ ^]^ the ECSA of PCS was calculated to be 867.464 cm^2^ mg^−1^ over 300 times higher than that of CNF LDH (2.254 cm^2^ mg^−1^) and FePc (2.729 cm^2^ mg^−1^). To investigate the relationship between electrocatalytic activity and ECSA, the specific activity of CNF LDH, PCS, and FePc were evaluated based on their ECSA values (Figure S15, Supporting Information). While PCS demonstrated commendable OER performance, the observed current density per unit geometrical area of the electrode was attributed primarily to its large ECSA rather than its intrinsic catalytic activity. Thus, it is anticipated that integrating PCS as a conductive substrate with a large active surface area can significantly enhance the OER and ORR performance of CNF LDH and FePc by maximizing their effective utilization.

To evaluate the bifunctional OER/ORR performance of the LDH||PCS||Pc nanocomposite catalyst, LSV curves were obtained for LDH||PCS||Pc and IrO_2_ with VC (denoted as IrO_2__VC, for OER) and Pt/C (for ORR) as reference catalysts (Figure 4d,e). The *E*_OER_ at 10 mA cm^−2^ for LDH||PCS||Pc was 1.51 V_RHE_, significantly lower than that of IrO_2__VC (1.74 V_RHE_). Meanwhile, the *E*_ORR_ at −3 mA cm^−2^ for LDH||PCS||Pc was comparable to that of the Pt/C (0.89 V_RHE_). Additionally, both LDH||PCS||Pc and Pt/C exhibited electron transfer numbers close to 4 (Figure S16, Supporting Information), indicating high selectivity for H_2_O formation. This was further supported by the Koutecky–Levich (K–L) plot, which showed an electron transfer number ranging from 3.98 to 4.01 across various applied potentials (Figure S17, Supporting Information). Moreover, we examined whether the increases in surface area and pore volume resulting from exfoliation contributed to enhanced electrochemical performance. The OER and ORR activities of CNF LDH and FePc showed negligible differences before and after HSE treatment, indicating that the increased surface area and porosity did not directly translate to improved electrocatalytic performance (Figure S18, Supporting Information). In contrast, although the LDH||PCS||Pc composite exhibited a relatively lower surface area and pore volume compared to pristine PCS, the two active 2D nanosheets (i.e., CNF LDH and FePc) were uniformly dispersed on the PCS substrate. This uniform distribution facilitated effective exposure of active sites and promoted a strong synergistic interaction, ultimately enhancing electrocatalytic activity.

To further elucidate the role of each component, PCS was individually combined with either CNF LDH or FePc, and their performances were compared with that of the LDH||PCS||Pc nanocomposite. Specifically, the OER performance of a catalyst prepared by mixing CNF LDH and PCS at a 1:1 mass ratio (denoted as LDH||PCS) was evaluated (Figure S19, Supporting Information), following the optimized catalyst design conditions established for LDH||PCS||Pc (detailed in Note S3, Figures S20 and S21, Supporting Information). The LDH||PCS catalyst exhibited OER performance comparable to that of LDH||PCS||Pc, indicating that FePc does not contribute to OER activity and confirming CNF LDH as the principal OER‐active component in the composite. Similarly, the catalyst formed by coupling FePc with PCS at a 1:1 mass ratio (denoted as PCS||Pc) displayed ORR activity similar to that of LDH||PCS||Pc (Figure S22a, Supporting Information), along with an electron transfer number close to 4 (Figure S22b, Supporting Information). In contrast, PCS alone exhibited a lower electron transfer number (≈3.6) in ORR, compared to FePc (Figures S12b and S13b, Supporting Information), further supporting the conclusion that FePc serves as the primary ORR‐active site in the LDH||PCS||Pc nanocomposite. Moreover, mass activities of electrocatalysts were evaluated to confirm the synergistic effect of the HSE method and the well‐engineered PCS substrate. Based on electrochemical analyses, CNF LDH and FePc were identified as the primary active materials for OER and ORR, respectively, in the LDH||PCS||Pc composite. According to the performance evaluation conditions, the mass loading of OER‐ and ORR‐active materials per unit electrode area was estimated to be 0.405 mg cm^−2^ for CNF LDH and FePc, and 0.135 mg cm^−2^ (i.e., one‐third of 0.405 mg cm^−2^) for LDH||PCS||Pc, LDH||PCS, and PCS||Pc. The OER potentials required to achieve a current density of 100 A g^−1^ (*E*_OER_ at 100 A g^−1^) were 1.52 V_RHE_ for LDH||PCS||Pc and 1.53 V_RHE_ for LDH||PCS, both significantly lower than that of CNF LDH_VC (1.57 V_RHE_), as shown in Figure S23 (Supporting Information). Similarly, the ORR mass activities at 0.7 V_RHE_ were −43.14 A g^−1^ for LDH||PCS||Pc and −42.95 A g^−1^ for PCS||Pc—both over three times higher than that of FePc_VC (−13.97 A g^−1^) (Figure S24, Supporting Information). The catalytic activity of each material was further evaluated by estimating the minimum turnover frequency (TOF), assuming that all metal elements in the active materials—CNF LDH for OER and FePc for ORR—function as active sites.^[^ ^12^ ^]^ The number of metal cations was quantified via ICP‐OES analysis (Table S2, Supporting Information). For both OER and ORR, the minimum TOF values increased when PCS was employed as the conductive substrate, measured at overpotentials of 350 and 380 mV, respectively (Figure S25, Supporting Information). This enhancement in minimum TOF was consistently observed in the LDH||PCS||Pc and LDH||PCS catalysts for OER, as well as in PCS||Pc for ORR. These improvements can be attributed to the synergistic effects of the high‐shear exfoliation (HSE) process, which increases nanosheet accessibility, and the use of PCS, which offers a high surface area and abundant anchoring sites for effective nanosheet integration.

In addition, we synthesized the LDH||VC||Pc using CNF LDH, FePc, and VC, a widely reported conductive substrate, to investigate the role of porous conductive substrate. VC showed a high BET surface area and pore volume, indicating its suitability as a comparison substrate (Table S1, Supporting Information). Subsequently, XRD confirmed the successful nanocomposite formation (Figure S26, Supporting Information). Notably, PCS provided a larger C_dl_ for the electrocatalyst compared to VC as observed in the LDH||VC||Pc composite (Figure S27, Supporting Information). This difference in ECSA explained the higher OER current density of LDH||PCS||Pc, even though the ORR performance of LDH||PCS||Pc and LDH||VC||Pc was similar. This similarity in ORR performance was likely due to the solubility limitation of oxygen gas in the electrolyte (Figure S28, Supporting Information). Additionally, electrochemical stability tests were performed on LDH||PCS||Pc, LDH||VC||Pc, as well as FePc and CNF LDH, which served as ORR‐ and OER‐active materials, respectively. In the ORR stability tests, all catalysts maintained stable electrochemical performance over 25 h at 0.4 V_RHE_—a potential sufficient for each catalyst to reach its limiting current density (Figure S29, Supporting Information). For OER, chronopotentiometry measurements were conducted at a current density of 10 mA cm^−2^. Under these conditions, LDH||VC||Pc and CNF LDH exhibited inferior stability compared to LDH||PCS||Pc (Figure S30, Supporting Information).

Inductively coupled plasma optical emission spectroscopy (ICP‐OES) analysis revealed that the electrolyte from the CNF LDH system contained significantly higher concentrations of dissolved metal cations than those from LDH||PCS||Pc and LDH||VC||Pc (Table S3, Supporting Information), suggesting greater material degradation. Complementary XRD analysis showed a marked reduction in the intensity of the (003) plane peak after OER for CNF LDH and LDH||VC||Pc, while LDH||PCS||Pc retained a clear (003) reflection—indicating better structural preservation (Figure S31, Supporting Information). These results imply that the absence of an appropriate substrate may lead to nanosheet detachment and metal ion leaching. Moreover, SEM images before and after OER revealed that LDH||PCS||Pc remained well‐attached to the substrate, in stark contrast to LDH||VC||Pc, which showed signs of detachment (Figure S32, Supporting Information). These findings collectively demonstrate that PCS serves as an effective porous conductive substrate, offering abundant anchoring sites and superior structural stability under electrochemical conditions. Furthermore, the potential gap (∆*E*) between the *E*_OER_ at 10 mA cm^−2^ and the *E*_ORR_ at −3 mA cm^−2^ for LDH||PCS||Pc was 0.63 V, smaller than that of reference catalysts (0.85 V) (Figure 4f). To the best of our knowledge, the ∆*E* of LDH||PCS||Pc is the lowest reported to date for oxygen bifunctional catalysts in alkaline electrolytes (Figure 4g; Table S4, Supporting Information).

### 2.5 LDH||PCS||Pc as Air Cathode for Zinc‐Air Battery Application

**Figure 5:**
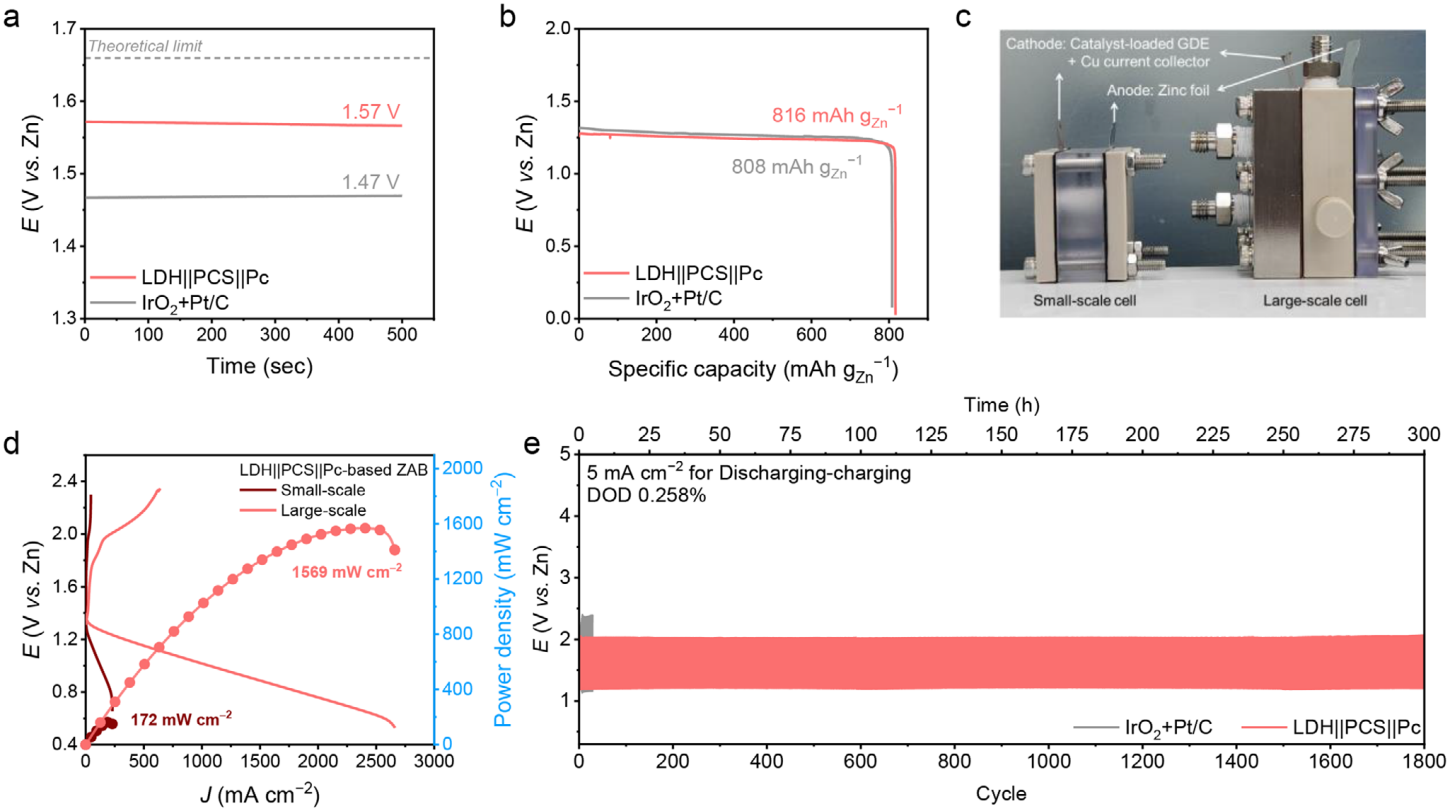
Electrochemical performance of the zinc‐air battery (ZAB) based on LDH||PCS||Pc catalyst. a) Open‐circuit potential measurement of the ZAB with LDH||PCS||Pc and IrO_2_+Pt/C air cathode. b) Specific capacity for LDH||PCS||Pc‐ and IrO_2_+Pt/C‐based ZAB cells. c) Photograph of the conventional ZAB and large‐scale ZAB. d) Polarization discharge–charge curves and power densities for LDH||PCS||Pc‐based small‐scale and large‐scale ZAB cells. e) Galvanostatic discharge–charge curves with 10 min cycles at a current density of 5 mA cm^−2^ for LDH||PCS||Pc‐ and IrO_2_+Pt/C‐based large‐scale ZAB cells.

A home‐assembled ZAB was constructed using polished zinc foil as the anode and a catalyst‐loaded gas diffusion electrode (GDE) as the air cathode to evaluate the electrochemical performance. Catalysts (LDH||PCS||Pc and IrO_2_+Pt/C mixture) were drop‐casted onto carbon paper gas diffusion media with a microporous layer at a loading of 1 mg cm^−2^. The LDH||PCS||Pc‐based ZAB exhibited an open‐circuit potential (OCP) of 1.57 V, surpassing the 1.47 V OCP of the IrO_2_+Pt/C‐based ZAB (**Figure** **5**a). The high OCP of the LDH||PCS||Pc‐based ZAB powered a fan, demonstrating its practical application potential (Figure S33 and Video S1, Supporting Information). Additionally, the LDH||PCS||Pc‐based ZAB achieved a maximum power density of 172 mW cm^−2^, comparable to the IrO_2_+Pt/C‐based ZAB (Figure S34, Supporting Information). Its specific capacity was 816 mAh g_Zn_^−1^, measured until complete consumption of the zinc electrode (Figure S35, Supporting Information), slightly exceeding the IrO_2_+Pt/C‐based ZAB's specific capacity of 808 mAh g_Zn_^−1^ (Figure 5b). The discharge potential of the LDH||PCS||Pc‐based ZAB was 1.27 V at 5 mA cm^−2^, decreasing to 1.12 V at 30 mA cm^−2^, and then returning to the initial discharge potential, indicating excellent rate capability (Figure S36, Supporting Information). The battery demonstrated remarkable long‐term durability under continuous discharge–charge cycling at 5 mA cm^−2^ (Figure S37, Supporting Information). The discharge–charge potential gap for the LDH||PCS||Pc‐based ZAB increased only slightly, from 0.71 to 0.84 V over 500 cycles, while the potential gap for the IrO_2_+Pt/C‐based ZAB increased significantly, from 0.74 to 1.27 V, within just 75 cycles. These results highlight the high efficiency, outstanding stability, and cost‐effectiveness of the LDH||PCS||Pc‐based ZAB system, which is based on earth‐abundant transition metal and carbon materials.

Furthermore, a large‐scale cell and system were designed to efficiently supply electrolyte and gaseous reactants, validating the practical feasibility of the LDH||PCS||Pc‐based ZAB with high efficiency and durability. The active area of the air cathode was expanded to 6.25 cm^2^, more than 3.5 times larger than the conventional small‐scale cell (1.767 cm^2^) (Figure 5c; Figure S38, Supporting Information). The gas‐diffusion channel of the large‐scale cell was designed based on previous studies demonstrating that optimized gas flow field shapes facilitate effective mass transfer of gaseous reactant into the catalyst layer.^[^ ^23^ ^]^ To address challenges such as zinc anode dendrite formation, electrode deformation, ion concentration gradient, and electrolyte evaporation, a flowing‐electrolyte system was incorporated into the large‐scale cell design (Figure S39, Supporting Information).^[^ ^1d^ ^]^ This system enabled the LDH||PCS||Pc‐based ZAB to achieve an extremely enhanced discharge–charge performance in large‐scale cell, compared to that of the small‐scale cell under the same operating condition (Figure 5d). Thanks to the well‐engineered system architecture, the large‐scale LDH||PCS||Pc‐based ZAB delivered a peak power density of 1569 mW cm^−2^, representing a 9.12‐fold improvement over the small‐scale system. To validate the reliability of the enhanced peak power density achieved in the optimized large‐scale ZAB system, we compared the LDH||PCS||Pc‐based cell with a reference system employing benchmark catalysts (IrO_2_ + Pt/C) and performed discharge tests under an Ar atmosphere. Additionally, the effectiveness of the gas flow field design used in this study was evaluated in terms of its ability to improve O_2_ transport to the catalyst layer on the gas diffusion electrode (GDE), as detailed in Note S4 and Figures S40–S43 (Supporting Information). The specific capacity and maximum capacity of the LDH||PCS||Pc‐based large‐scale ZAB cell were estimated to be 817 mAh g_Zn_^−1^ and 1009 mAh, respectively, based on the mass of zinc consumed during discharge (Figures S44 and S45, Supporting Information). Notably, the specific capacity not only matched that of the small‐scale ZAB cell but also closely approached the theoretical maximum specific capacity of ZABs (≈820 mAh g_Zn_^−1^),^[^ ^24^ ^]^ highlighting the effectiveness of the optimized system design. Additionally, the LDH||PCS||Pc‐based large‐scale ZAB exhibited outstanding stability. The difference in the discharge–charge voltage gap between the initial cycle (0.861 V at 0 h, 1st cycle) and the final cycle (0.863 V at 300 h, 1800th cycle) was almost negligible during continuous operation at a discharge–charge current density of 5 mA cm^−2^ for 300 h (Figure 5e; Figure S46, Supporting Information). To robustly validate the stability of the LDH||PCS||Pc‐based large‐scale ZAB cell, discharge–charge cycling tests were performed at progressively increasing current densities of 5, 10, and 20 mA cm^−2^, with each step maintained for 25 h (150 cycles). Notably, after operating at elevated current densities for a total of 75 h, the current density was reduced back to 5 mA cm^−2^. At this point, the discharge–charge voltage gap closely matched that observed during the initial 5 mA cm^−2^ step (Figure S47, Supporting Information). These results clearly demonstrate the excellent durability and electrochemical stability of the LDH||PCS||Pc‐based large‐scale ZAB cell, even under harsh operating conditions involving prolonged cycling and high current densities. In contrast, the IrO_2_+Pt/C‐based ZAB rapidly degraded, losing its initial potential gap after just a few cycles under a discharge–charge current density of 5 mA cm^−2^. Furthermore, to clearly demonstrate the stability of the battery system, we specified the depth of discharge (DOD) during galvanostatic discharge–charge cycling tests. Based on the maximum capacity of 1000 mAh for the large‐scale ZAB cell, the DOD values corresponding to discharge current densities of 5, 10, and 20 mA cm^−2^ were calculated to be 0.258%, 0.516%, and 1.032%, respectively. These values were derived under a cycling protocol of 10 min per cycle (5 min discharge followed by 5 min charge). These results highlight the significant performance enhancement achievable through the combination of an efficient oxygen bifunctional electrocatalyst with a well‐designed system architecture, underscoring the potential of LDH||PCS||Pc‐based ZABs for large‐scale practical applications.

## 3 Conclusion

In summary, we have successfully developed a novel bifunctional electrocatalyst by integrating OER‐active CNF LDH and ORR‐active FePc onto a highly conductive PCS using the HSE method. This one‐step process simultaneously exfoliates and integrates materials, simplifying fabrication and paving the way for scalable production. PCS, characterized by its excellent electrical conductivity and abundant anchoring sites, enabled strong binding with active materials, as corroborated by DFT calculations and EXAFS analysis. As a result, the LDH||PCS||Pc nanocomposite exhibited exceptional bifunctional catalytic activity, achieving a remarkably low potential gap (Δ*E* = 0.63 V) between OER and ORR. An aqueous ZAB constructed using LDH||PCS||Pc showed a high‐power density and exceptional cycling stability, even under high current density conditions. This study highlights a practical and scalable approach for integrating multiple catalytic components, offering significant potential for advancing high‐performance, multifunctional electrocatalysts in diverse electrochemical energy conversion and storage applications.

## 4 Experimental Section

### Materials

Cobalt nitrate hexahydrate (Co(NO_3_)_2_·6H_2_O, ≥99.0%), iron nitrate nonahydrate (Fe(NO_3_)_3_·9H_2_O, ≥99.0%), nickel nitrate hexahydrate (Ni(NO_3_)_2_·6H_2_O, ≥99.0%), zinc nitrate hexahydrate (Zn(NO_3_)_2_·6H_2_O, ≥99.0%), 2‐methylimidazole (Im, 99%), iron phthalocyanine (FePc, dye content ≈90%), sodium hydroxide (NaOH, ≥98.0%), hydrochloric acid, (HCl, 35.0–37.0%), nitric acid (HNO_3_, 70%), IrO_2_ (99.9%), and Pt/C (20 wt.% loading) were purchased from Sigma–Aldrich and were used without further purification.

### Synthesis of PCS

Typically, Im (7.882 g, 96 mmol) was dissolved in 80 mL methanol with stirring 10 min in flask A. Zn(NO_3_)_2_·6H_2_O (3.570 g, 12 mmol) and Co(NO_3_)_2_·6H_2_O (3.492 g, 12 mmol) were dissolved in 240 mL methanol with stirring 10 min in flask B. Then, flask B was subsequently added into flask A with vigorous stirring for 24 h at room temperature. The obtained Zn and Co combined ZIF (C_ZIF) containing solution was washed with deionized water and ethanol three times and finally dried at 70 °C. The prepared powder was then transferred into a ceramic boat and placed in a tube furnace. The sample was heated to 900 °C with a heating rate of 10 °C min^−1^ and kept at 900 °C for 1 h under flowing N_2_ gas and then naturally cooled to room temperature. To remove aggregated bulk cobalt particles, acid etching in aqua regia (a mixture of HCl and HNO_3_ at a volume ratio 3:1) was employed at 40 °C for 24 h, followed by heating the resulting solids at 900 °C for 1 h under flowing N_2_ gas.

### Synthesis of CNF LDH

Bulk CNF LDH was synthesized by the co‐precipitation method. In a typical process, Ni(NO_3_)_2_·6H_2_O (8.724 g, 30 mmol), Fe(NO_3_)_3_·9H_2_O (4.040 g, 10 mmol), and Co(NO_3_)_2_·6H_2_O (1.164 g, 4 mmol) were dissolved in 500 mL deionized water with magnetic stirring. Simultaneously, 1 м NaOH solution was added dropwise under vigorous stirring until pH ≈10. The obtained mixture was stirred for 24 h at room temperature. The CNF LDH‐containing suspension was washed with deionized water and ethanol three times and finally dried at 70 °C.

### Synthesis of LDH||PCS||Pc

LDH||PCS||Pc was synthesized by the high‐shear exfoliation (HSE) method. 1 g of PCS was dispersed in 500 mL of deionized water via ultrasonication for 10 min to form a clear black suspension. Then, 1 g of bulk CNF LDH and 1 g of bulk FePc were incorporated into the PCS suspension. The suspension was homogenized in an Ultra‐Turrax (IKA) at 10 000 rpm for 30 min. After HSE method, the obtained product was rinsed with deionized water and ethanol three times and finally dried at 70 °C under vacuum overnight. Mass ratio‐controlled samples (i.e., LDH_(1.5)_||PCS||Pc_(0.5)_ and LDH_(0.5)_||PCS||Pc_(1.5)_), LDH||VC||Pc, LDH||PCS, and PCS||Pc were synthesized using the same procedures as LDH||PCS||Pc.

### Material Characterization

The crystalline structures were characterized via X‐ray diffraction (XRD) analysis employing a D8 ADVANCE X‐ray diffractometer (Bruker AXS) with Cu Kα radiation (λ = 1.5406 Å). Morphological features and elemental distribution of the samples were investigated using high‐resolution transmission electron microscopy (HR‐TEM) on a JEOL JEM‐2100F instrument operated at an accelerating voltage of 200 kV. SEM images were obtained using an SU7000 field emission scanning electron microscope (Hitachi High‐Tech Corporation). Compositional analysis was performed via inductively coupled plasma optical emission spectrometry (ICP‐OES) using a Varian 700‐ES spectrometer. Atomic force microscopy (AFM) measurements were conducted on a Multimode 8 system (Veeco) utilizing tapping mode in ambient conditions. Raman spectra were collected from five different sites with an exposure of 1 s and accumulation of 150 times for each sample using an Alpha 300s micro‐Raman spectrometer (WITec, Germany). The excitation wavelength and laser power were 532 nm and 0.5 mV, respectively. X‐ray photoelectron spectroscopy (XPS) studies were carried out on a Thermo‐Fisher Scientific ESCALAB 250XI system equipped with a monochromatic Al Kα X‐ray source. All XPS spectra were calibrated with reference to the predominant C─C binding energy at 284.8 eV. The BET surface area, pore volume, and pore diameter of the obtained samples were measured on an ASAP 2023 (Micromeritics Instrument Corp., USA) with flowing N_2_ gas after degassing pretreatment at 80 °C for 15 h. XAS was performed at the beamline 6D of Pohang Accelerator Laboratory. The incident beam was filtered by a Si (111) double crystal monochromator and detuned by 30% to remove high‐order harmonics. The incident photon energy was then calibrated using a standard reference metal. The powder sample was pressed using a hand‐pelletizer to the desired thickness so that the X‐ray beam could pass through a large enough number of atoms. Background removal and normalization of XAS spectra were conducted using the Athena software. Fourier transform of EXAFS spectra was carried out using the Artemis software to obtain coordination numbers and interatomic distances.

### Electrochemical Measurements

The electrochemical performances and properties of the catalysts were measured using a potentiostat electrochemical analyzer (nStat, Ivium Technologies). A three‐electrode system was used for the measurements: Hg/HgO as the reference electrode (Re‐61AP, ALS), Pt wire as the counter electrode, and a catalyst‐loaded glassy carbon RDE as the working electrode. A catalyst ink was prepared by dispersing 10 mg of catalyst and 2 mg of Vulcan carbon (XC‐72, Cabot) in 980 µL of anhydrous ethanol with 20 µL of Nafion solution (5 wt.%, Aldrich). The catalyst ink was well homogenized in a sonication bath for 1 h. To preparing the working electrode, 10 µL of ink was loaded on the glass carbon RDE (Area: 0.247 cm^2^) and fully dried at 50 °C. The electrochemical OER analyses were conducted in Ar‐saturated 1 м KOH (pH 14) and electrochemical ORR analyses were measured in O_2_‐saturated 0.1 м KOH (pH 13). The measured potential versus Hg/HgO was converted to the RHE using the following equation:

(2) $$E\left(\mathrm{vs}.\mathrm{RHE}\right)=E\left(\mathrm{vs}.\mathrm{Hg}/\mathrm{HgO}\right)+0.0592\times \mathrm{pH}+{E^{o}}_{\mathrm{re}f,\mathrm{Hg}/\mathrm{HgO}}$$

(*E*⁰_ref, Hg/HgO_ = 0.118 V vs. normal hydrogen electrode (NHE) at 25 °C)

Cyclic voltammetry was performed in the range of 0.2–1.0 V versus RHE for 20 cycles at a scan rate of 100 mV s^−1^ to clean the surface of the working electrode before electrochemical analyses. For OER activity measurements, the LSV curves were measured in the potential range of 1.0–1.9 V versus RHE at a scan rate of 10 mV s^−1^ and an electrode rotating speed of 1600 rpm. To compare the ECSA of each catalyst, the C_dl_ was measured in Ar‐saturated 1 м KOH electrolyte in the range of 0.75 V–0.85 V versus RHE (non‐Faradaic region) at the scan rates of 5, 10, 20, 40, 60, 80, 100, and 120 mV s^−1^. For the ORR activity measurements, RRDE (PINE Research Instrumentation Inc.) measurement was conducted for identifying the electron transfer number of ORR. Pt (Area: 0.1866 cm^2^) ring was electrochemically cleaned in the range of 0.05–1.2 V versus RHE in Ar‐saturated 0.1 м KOH at the scan rate of 500 mV s^−1^ for 50 cycles. Before measuring the ORR activity, O_2_ gas was purged into the electrolyte for 3 min to minimize the time interval between Pt ring cleaning and ORR activity measurement for preventing the surface passivation of the Pt ring. The LSV curves were measured in the potential range of 1.1–0.2 V versus RHE at a scan rate of 10 mV s^−1^ and an electrode rotating speed of 1600 rpm. Further, the potential of Pt ring was fixed at 1.3 V versus RHE to oxidize the hydrogen peroxide (H_2_O_2_) which was produced from 2e^−^ reaction pathway. The electron transfer number was calculated using the following relation:

(3) $$\mathrm{Electron}\;\mathrm{transfer}\;\mathrm{number}\;\left(n\right)=\frac{4\times i_{d}}{i_{d}\times \frac{i_{r}}{N}}$$

where *i*_d_, *i*_r_, and *N* are the disk current, ring current, and collection efficiency, respectively. The collection efficiency was estimated using the [Fe(CN)_6_]^3−/4−^ redox system.^[^ ^25^ ^]^ Chronoamperometry was conducted at −0.3 V versus Ag/AgCl (RE‐1B, ALS) for 50 s while the ring potential was fixed at 0.5 V versus Ag/AgCl in Ar‐saturated 0.1 м KOH + 2 mм K_3_[Fe(CN)_6_] (Figure S48, Supporting Information). The background ring current (*i*_r,bg_) was measured by a similar method with disk potential of 0.5 V versus Ag/AgCl. The collection efficiency is expressed as

(4) $$\mathrm{Collection}\;\mathrm{efficiency}\;\left(N\right)=\frac{\left|i_{r}-i_{r,\;bg}\right|}{i_{d}}$$

The calculated collection efficiency was 37.58%, which was similar to the provided value from the manufacturer (37%). The K‐L analysis was conducted by LSV curves, which were obtained at different electrode rotation speeds of 400, 900, 1600, and 2500 rpm. K‐L plot depicts the relationship between the current density, electrode rotation speed, and electron transfer number according to the equation below:

(5) $$\frac{1}{i}=\frac{1}{i_{k}}+\frac{1}{0.62nFD_{0}^{2/3}\omega^{1/2}v^{-1/6}C_{0}}$$

where *i, i_k_, n, F, A, D_0_, ω* (= 2πN, N is the linear rotation speed)*, v*, and *C_0_* denote the measured current, kinetic current, electron transfer number, Faraday constant (96485 C mol^−1^), diffusion coefficient of O_2_ in the electrolyte at 298 K (1.9 × 10^−5^ cm^2^ s^−1^), rotation speed (rad s^−1^), kinematic viscosity of O_2_ (0.89 × 10^−2^ cm^2^ s^−1^), and concentration of O_2_ in bulk (1.2 × 10^−6^ mol cm^−2^), respectively.^[^ ^26^ ^]^

The turnover frequency (TOF) value was calculated from the equation below:

(6) $$\mathrm{TOF}\;=\frac{J\;\times A}{4\;\times F\;\times m}$$

where J, A, F, and m are the current density at a given overpotential, area of electrode, Faraday constant (96485 C mol^−1^), and the number of moles of metals contained in active materials for each electrochemical reaction on the electrode.

### Zinc‐Air Battery (ZAB) Demonstration

The electrochemical properties of ZAB were investigated by a home‐made cell. The prepared catalyst ink was drop‐casted on carbon paper (Sigracet 39BB, SGL Carbon) as an air cathode with a loading of 1 mg cm^−2^. A polished zinc foil (99.98%, Alfa Aesar) was applied as a metal anode, and 6 m KOH + 0.2 m zinc acetate aqueous solution was used as the electrolyte. The specific capacities (unit as mAh g_Zn_^−1^) were obtained by using the galvanostatic discharge profiles at a consistent rate of 5 mA cm^−2^ normalized to the consumed mass of zinc, using the following equation^[^ ^27^ ^]^:

(7) $$\mathrm{Specific}\;\mathrm{capacity}\;=\frac{i_{app}\times t}{m_{Zn}}$$

where *i*_app_, *t*, and *m*_Zn_ are the applied current (A), the discharge time (h), and total weight of the consumed zinc (g), respectively. Notably, peristaltic pump (JWTE600, JenieWell) was conducted while demonstrating the large‐scale ZAB with electrolyte flow system. The flow rate of electrolyte was fixed at 10 mL min^−1^, while the gas flow rate was controlled to 300 or 100 mL min^−1^ using a flow meter.

The depth of discharge (DOD) was estimated according to the equation:

(8) $$\mathrm{DOD}\;\left(\%\right)=\frac{i_{app}\times t}{Q_{total}}\;\times 100$$

where *i*_app_, *t*, and Q*_total_* are the applied current (mA), the discharge time per cycle (h), and maximum capacity (mAh), respectively.

### Computational Method

Density functional theory using the Vienna ab‐initio simulation package (VASP) was performed.^[^ ^28^ ^]^ The Perdew‐Burke‐Ernzerhof (PBE) functional was used to describe the exchange‐correlation of valence electron.^[^ ^29^ ^]^ The cut‐off energy was set to 400 eV for PCS‐FePc structure, and 520 eV for PCS‐CNF LDH structure. The projector augmented method was utilized.^[^ ^30^ ^]^ The Brillouin zone was sampled using a 100 k‐points density per Å^−3^ in the reciprocal lattice. The valence state file (POTCAR) of each element was selected as following: Fe_pv for Fe, Co for Co, Ni_pv for Ni, H for H, C for C, N for N, and O for O. To mimic reasonable alloy process, the special quasi‐random structures (SQS) method^[^ ^31^ ^]^ using the *mcsqs* code provided in the Alloy Theoretic Automated Toolkit (ATAT) was used.^[^ ^32^ ^]^ All pristine atomic structures were extracted from the Crystallography Open Database^[^ ^33^ ^]^ and were displayed using VESTA.^[^ ^34^ ^]^

## Conflict of Interest

The authors declare no conflict of interest.

## Supporting information



Supporting Information

Supporting Video

## Data Availability

The data that support the findings of this study are available from the corresponding author upon reasonable request.
