# Supplementary material for: Enhanced Bifunctional Electrocatalysis for Zinc‐Air Battery Using Porous Conductive Substrate with Abundant Anchoring Sites
Source: Adv Sci (Weinh). 2025 Jul 28;12(40):e06172. doi: 10.1002/advs.202506172 (PMC12561423; doi:10.1002/advs.202506172)
Supplement: Supplementary file 1 — Supporting Information [file ADVS-12-e06172-s001.docx]

Supporting Information

**Enhanced Bifunctional Electrocatalysis for Zinc-Air Battery Using Porous Conductive Substrate with Abundant Anchoring Sites**

*Jongkyoung Kim, Je Min Yu, Jun-Yong Choi, Seong-Hun Lee, Han Uk Lee, Dongrak Oh, Hyunju Go, Wonsik Jang, Seunghyun Lee, Jaewon Cho, Sung Beom Cho, Tae Joo Shin, Hyunjoo Lee, Sang-Goo Lee, Ji-Wook Jang*, Seungho Cho*, and Wook Jo**

**Supporting Note 1: Role of Zn and Co in the thermal conversion of Zeolitic Imidazolate Framework**

Zeolitic imidazolate framework (ZIF), a well-known subclass of metal-organic frameworks (MOF), can be formed through the coordination of metal ions with imidazolate linker.^[1]^ ZIF-8 and ZIF-67 exhibit isostructural characteristics, represented by the general formula [M(MeIm)_2_]_n_ (where M = Zn and Co, respectively), with nearly identical unit cell parameters (ZIF-8: a = b = c = 16.9910 Å; ZIF-67: a = b = c = 16.9589 Å). This structural similarity enables the systematic design of a combined ZIF (denoted as C_ZIF) featuring homogeneously distributed metal centers. The successful formation of the ZIF phase was confirmed by XRD analysis, as evidenced in Figure S1.

Both Zn^2+^ and Co^2+^ ions readily form sodalite-type frameworks when coordinated with 2-methylimidazole.^[2]^ The strategic incorporation of Zn²⁺ ions can facilitate the selective substitution of Co^2+^ positions, thereby enabling precise control over the spatial distribution of Co^2+^ ions. This controlled substitution mechanism plays a crucial role in the subsequent formation of well-dispersed Co nanoparticles. The introduction of Zn serves two key purposes: it enables to expand Co-Co interatomic distances and generate abundant nitrogen (N) coordination sites, ultimately enhancing the stability of the resulting Co nanoparticles.

During thermal treatment under inert conditions, Zn atoms volatilize due to their relatively low boiling point, while the organic linkers decompose to form nitrogen-doped porous carbon.^[3]^ Simultaneously, Co nodes undergo reduction through the carbonization process. This strategic design effectively suppresses Co-Co aggregation during high-temperature treatment, with the remaining Co atoms anchored on the N-doped carbon framework after Zn volatilization.

**Supporting Note 2: Scalable production of exfoliated 2D materials by high-shear exfoliation**

Exfoliation of 2D materials has been extensively studied to harness their unique physicochemical properties, which arise from their high aspect ratios and large surface areas. However, the practical application of these materials remains limited, as many exfoliation methods suffer from poor scalability due to low yield, long processing times, and high energy consumption. Therefore, efficient and scalable exfoliation methods are crucial.

High-shear exfoliation (HSE) has emerged as a promising exfoliation method for industrial-scale production of 2D materials, enhancing exfoliation efficiency and scalability.^[4]^ The shear exfoliation process requires two fundamental mechanical forces: a normal force to expand interlayer spacing and a lateral force to facilitate layer separation. In turbulent flow conditions, these forces manifest as pressure fluctuations (normal force) and velocity gradients (shear stress) within the fluid.^[5]^ Especially, the shear stress acts along the lateral direction of the layers and breaks an electrostatic interaction between adjacent layers. Furthermore, high-velocity fluid dynamics generate robust random collisions and multidirectional stresses, providing sufficient force to overcome electrostatic bonds between the adjacent layers. As a result, the HSE method enables a facile and scalable liquid-phase exfoliation process with a short processing time (~30 min) (Figure S2).

**Supporting Note 3: Investigation of the relationship between the component ratio and the electrochemical activity of LDH||PCS||Pc nanocomposite catalyst.**

To compare with the optimized LDH||PCS||Pc nanocomposite catalyst (with a CNF LDH:PCS:FePc mass ratio of 1:1:1), we fixed the mass ratio of electrochemically active materials to the conductive substrate at 2:1. Based on this ratio, two composite catalysts were synthesized with varying proportions of CNF LDH and FePc at 1.5:0.5 and 0.5:1.5, denoted as LDH_(1.5)_||PCS||Pc_(0.5)_ and LDH_(0.5)_||PCS||Pc_(1.5)_, respectively, following the same synthesis procedure. This comparison aimed to evaluate the effect of the relative ratio between the OER- and ORR-active materials.

OER activity measurements revealed that both LDH_(1.5)_||PCS||Pc_(0.5)_ and LDH_(0.5)_||PCS||Pc_(1.5)_ exhibited lower performance than the optimized LDH||PCS||Pc catalyst (Figure S20a). The reduced OER activity of LDH_(0.5)_||PCS||Pc_(1.5)_ was attributed to the decreased CNF LDH content, leading to fewer OER-active sites. Interestingly, although LDH_(1.5)_||PCS||Pc_(0.5)_ contained a higher amount of CNF LDH, its OER performance also declined, especially at high oxidation potentials. This decline was likely due to insufficient anchoring of exfoliated CNF LDH nanosheets on the limited anchoring sites of PCS during the HSE process, resulting in restacking of unanchored nanosheets and decreased accessibility of active sites. This hypothesis is supported by XRD patterns, where LDH_(1.5)_||PCS||Pc_(0.5)_ exhibited a sharper and more intense (003) plane peak, indicative of higher nanosheet stacking order (Figure S21). These findings suggest that insufficient anchoring between PCS and CNF LDH not only limits active site exposure but also diminishes intrinsic activity, implying that excessive CNF LDH loading may negatively impact OER performance. In contrast, the LDH||PCS||Pc and LDH_(0.5)_||PCS||Pc_(1.5)_ catalysts exhibited comparable ORR performance, with required potentials of 0.87 V and 0.86 V at −3 mA cm⁻^2^, respectively (Figure S20b). Both catalysts also demonstrated electron transfer numbers close to 4, confirming similar ORR activity (Figure S20c). However, the LDH_(1.5)_||PCS||Pc_(0.5)_ catalyst showed decreased ORR performance at −3 mA cm⁻^2^, which can be attributed to insufficient ORR active sites due to the lower FePc content. Additionally, the low FePc loading resulted in partial involvement of PCS in ORR, leading to a reduced electron transfer number.

Overall, these results confirm that the composite catalyst with a balanced 1:1:1 mass ratio of CNF LDH, PCS, and FePc delivers superior bifunctional OER/ORR performance.

**Supporting Note 4: Verification of high peak power density of well-designed LDH||PCS||Pc-based large-scale ZAB cells**

First, we constructed a ZAB cell using a benchmark air cathode catalyst composed of an IrO₂ + Pt/C mixture (1:1 mass ratio), a widely used reference for bifunctional activity. This mixture exhibited OER/ORR activity comparable to that of the LDH||PCS||Pc catalyst, demonstrating its applicability in ZAB systems (Figure S40, Supporting Information). Based on the electrochemical performance, the IrO₂ + Pt/C-based large-scale ZAB cell achieved a peak power density of 1611 mW cm⁻^2^, similar to that of the LDH||PCS||Pc-based system (Figure S41a). However, the lower OER activity of IrO₂ compared to CNF LDH resulted in inferior charging performance, a trend also observed in small-scale ZAB systems (Figure S34). These results indicate that the high peak power density is primarily attributed to the well-optimized cell architecture of the large-scale ZAB system, rather than to catalyst performance alone. The reproducibility of the high power output from the LDH||PCS||Pc-based large-scale ZAB was confirmed by repeated discharge measurements across three independent devices (Figure S41b). Given that discharge performance in ZABs is directly governed by ORR activity at the air cathode, we further examined whether the high performance of the LDH||PCS||Pc-based system stemmed solely from ORR activity. Comparative discharge tests were conducted under O₂ and Ar atmospheres. Under Ar, the discharge current was negligible compared to that under O₂, confirming that the observed power output is derived from the ORR activity of the air cathode catalyst (Figure S41c). To evaluate the effect of gas flow field design, we compared the optimized interdigitated flow field with the commonly used serpentine configuration. The serpentine design enables lateral gas transport through interconnected channels, with vertical gas diffusion to the GDE driven solely by pressure gradients (Figure S42a). In contrast, the interdigitated design consists of alternating inlet and outlet channels separated by walls, which forces gas to penetrate vertically through the electrode before exiting (Figure S42b). At the standard O₂ flow rate of 300 mL min⁻^1^, both flow field configurations resulted in comparable peak power densities: 1569 mW cm⁻^2^ for the interdigitated design and 1530 mW cm⁻^2^ for the serpentine design. However, under limited O₂ supply (100 mL min⁻¹), the serpentine-based ZAB exhibited a sharp performance drop (1256 mW cm⁻^2^), whereas the interdigitated configuration maintained a high peak power density (1510 mW cm⁻^2^) (Figure S43). These results demonstrate that the interdigitated gas flow field more effectively facilitates O₂ transport to the catalyst layer, particularly under low-pressure conditions, thereby enhancing overall ZAB performance.

Figure S1. X-ray diffraction patterns of C_ZIF.


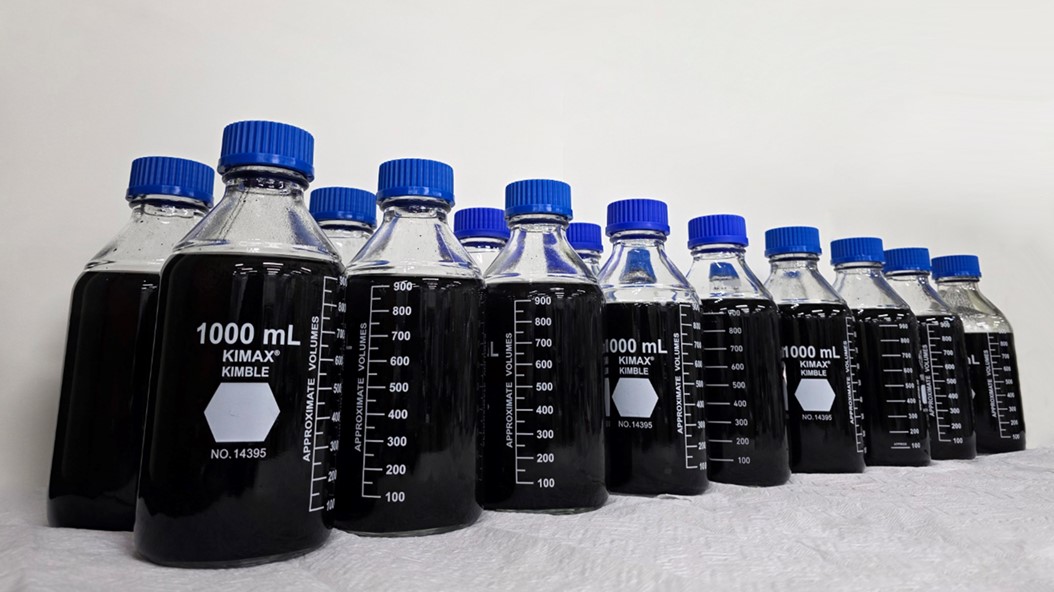


**Figure S2.** Photograph of LDH||PCS||Pc suspension produced by high-shear exfoliation process.

Figure S3. a) The modeling method of PCS atomic structure. b) The atomic structure of each component. c) Total radial distribution function (RDF) of Co, Ni, and Fe in PCS, CNF LDH, and bulk FePc, respectively. Dominant peaks are labeled with the specific atomic pairs and their interatomic distances.

Figure S4. a) AFM image and b) height profile of CNF LDH nanosheet. c) Photograph of the colloidal suspensions of CNF LDH nanosheets, exhibiting the Tyndall effect.

**
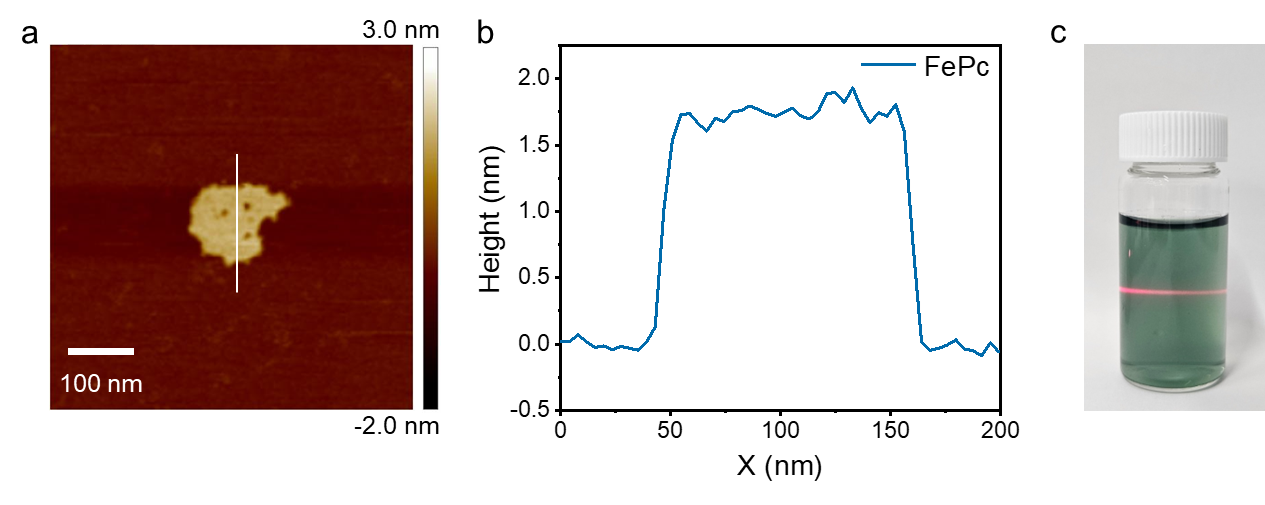
**

Figure S5. a) AFM image and b) height profile of FePc nanosheet. c) Photograph of the colloidal suspensions of FePc nanosheets, exhibiting the Tyndall effect.

Figure S6. Raman spectrum of FePc.

**Figure S7.** Nitrogen adsorption and desorption isotherms and pore size distribution curves (inset) obtained by BJH method for each sample.

**Table S1.** BET surface areas, pore volumes, and average pore diameters of different catalysts obtained by nitrogen adsorption and desorption measurements.

| Sample | BET surface area  (m^2^ g^−1^) | Pore volume  (cm^3^ g^−1^) | Average pore size  (nm) |
| --- | --- | --- | --- |
| CNF LDH | 78.8399 | 0.071667 | 3.63606 |
| CNF LDH after HSE | 160.3401 | 0.179408 | 4.47569 |
| PCS | 356.5117 | 0.320061 | 3.59103 |
| PCS after HSE | 357.6956 | 0.395633 | 4.42424 |
| FePc | 7.5005 | 0.036746 | 19.59673 |
| FePc after HSE | 9.4227 | 0.062054 | 26.34215 |
| LDH\|\|PCS\|\|Pc | 160.3743 | 0.277705 | 6.92643 |
| Vulcan carbon | 215.7024 | 0.395577 | 7.33561 |

**
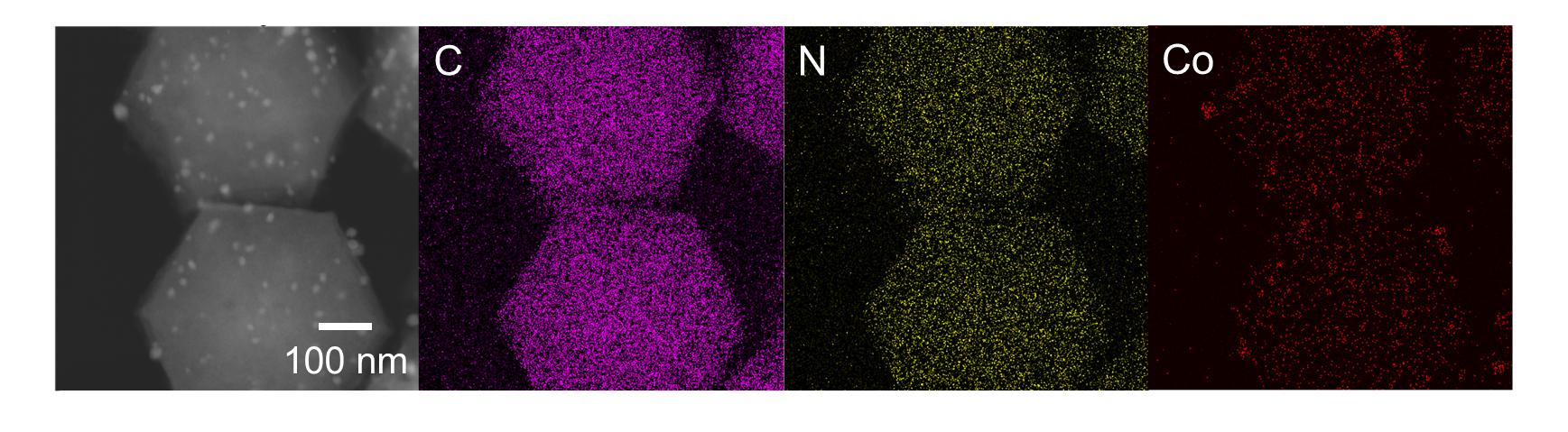
**

Figure S8. TEM image and corresponding element mappings of PCS.

**
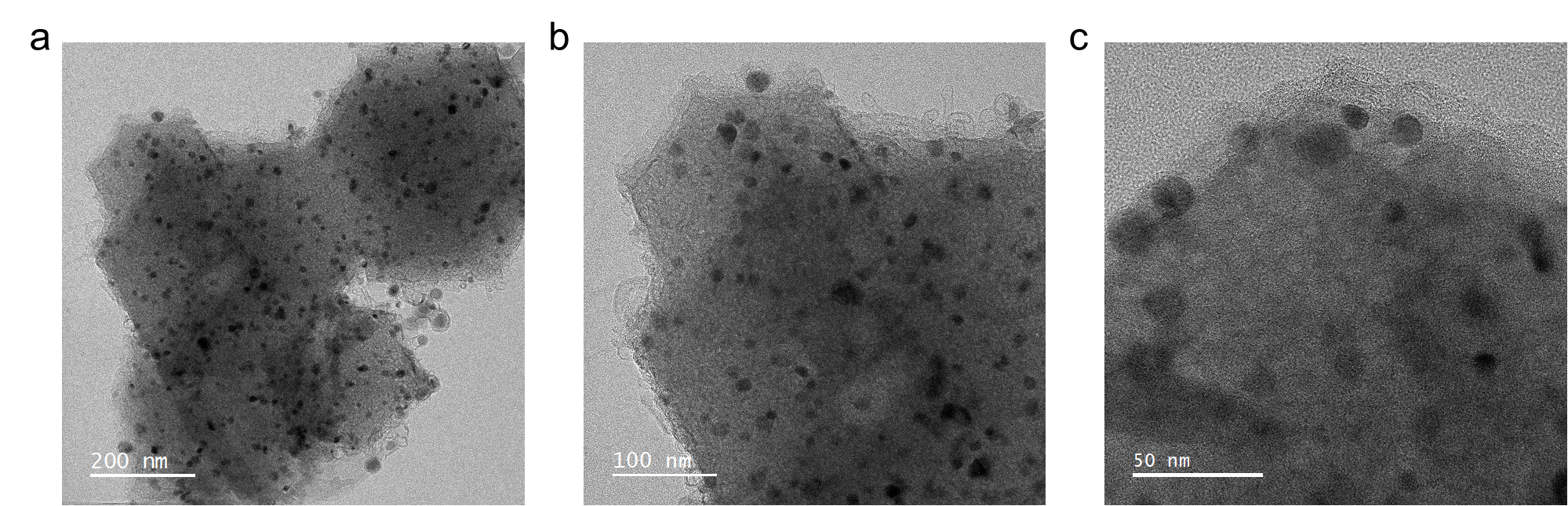
**

Figure S9. TEM images of LDH||PCS||Pc with different scales of a) 200 nm, b) 100 nm and c) 50 nm.

Figure S10. Particle size distribution of LDH||PCS||Pc.

Table S2. ICP-OES analysis of FePc, CNF LDH, PCS and LDH||PCS||Pc.

| Sample | Ni (wt. %) | Co (wt. %) | Fe (wt. %) |
| --- | --- | --- | --- |
| FePc | - | - | 9.93 |
| CNF LDH | 36.41 | 4.12 | 12.08 |
| PCS | - | 23.21 | - |
| LDH\|\|PCS\|\|Pc | 11.69 | 8.97 | 7.01 |

Figure S11. LSV curves of CNF LDH, PCS, and FePc for evaluating OER performance a) without and b) with VC.

Figure S12. a) LSV curves for evaluating ORR performance and b) Electron transfer number determined by rotating-ring disk electrode (RRDE) method of CNF LDH, PCS, and FePc

Figure S13. a) LSV curves for evaluating ORR performance and b) Electron transfer number determined by RRDE method for CNF LDH, PCS, and FePc with VC

Figure S14. CV curves of a) CNF LDH, b) PCS, and c) FePc to determine the double-layer capacitance (C_dl_) in 1 м KOH electrolyte. CV was conducted in the non-Faradaic regions of the potential at different scan rates of 5, 10, 20, 40, 60, 80, 100, and 120 mV s^−1^.

Figure S15. a) ECSA per unit mass of loaded-catalysts on the electrode of PCS, CNF LDH, and FePc. b) LSV curves for evaluating specific activities for CNF LDH, PCS, and FePc by considering the ECSA.

**Figure S16.** Electron transfer numbers determined by RRDE method for LDH||PCS||Pc and Pt/C.

Figure S17. a) LSV curves of LDH||PCS||Pc for ORR at different electrode rotation speeds. b) Koutecky-Levich (K-L) plots of LDH||PCS||Pc at different potentials.

**Figure S18.** LSV curves for determining a) OER activity of CNF LDH and b) ORR activity of FePc before and after HSE.

**Figure S19.** LSV curves of LDH||PCS and LDH||PCS||Pc for evaluating OER performance.

**Figure S20.** Comparison of LSV curves for determining a) OER and a) ORR electrochemical activity of LDH||PCS||Pc, LDH_(0.5)_||PCS||Pc_(1.5)_, and LDH_(1.5)_||PCS||Pc_(0.5)_. c) Electron transfer numbers determined by RRDE method for LDH||PCS||Pc, LDH_(0.5)_||PCS||Pc_(1.5)_, and LDH_(1.5)_||PCS||Pc_(0.5)_.

**Figure S21.** a) XRD patterns of LDH||PCS||Pc, LDH_(1.5)_||PCS||Pc_(0.5)_, and LDH_(0.5)_||PCS||Pc_(1.5)_. b) Close-up view of low-angle regions for the nanocomposites.

**Figure S22.** a) LSV curves for evaluating ORR performance and b) electron transfer numbers determined by RRDE method for PCS||Pc and LDH||PCS||Pc.

**Figure S23.** Comparison of the a) OER mass activities and b) required potential at 100 A g^−1^ of CNF LDH (with and without VC), LDH||PCS, and LDH||PCS||Pc.

**Figure S24.** Comparison of the a) ORR mass activities and b) current densities at 0.7 V_RHE_ of FePc (with and without VC), PCS||Pc, and LDH||PCS||Pc.

**Figure S25.** Minimum turnover frequency (TOF) of CNF LDH with VC, LDH||PCS, and LDH||PCS||Pc for OER and FePc with VC, PCS||Pc, and LDH||PCS||Pc for ORR. All TOF values were calculated from the current densities at overpotential 350 and 380 mV.

**Figure S26.** X-ray diffraction patterns of LDH||VC||Pc, FePc, CNF LDH and VC.

Figure S27. CV curves of a) LDH||PCS||Pc, and b) LDH||VC||Pc to determine the C_dl_ in 1 м KOH electrolyte. b) Comparison of C_dl_ of LDH||PCS||Pc. CV was conducted in the non-Faradaic regions of the potential at different scan rates of 5, 10, 20, 40, 60, 80, 100, and 120 mV s^−1^.

Figure S28. LSV curves of LDH||PCS||Pc, LDH||VC||Pc, IrO_2__VC, and Pt/C for evaluating a) OER and b) ORR performance.

**Figure S29.** Chronoamperometry measurement for 25 h at an applied potential of 0.4 V_RHE_ for the FePc, LDH||VC||Pc, and LDH||PCS||Pc.

**Figure S30.** Chronopotentiometry measurement for 25 h at an applied current density of 10 mA cm^−2^ for the CNF LDH, LDH||VC||Pc, and LDH||PCS||Pc.

**Table S3.** ICP-OES analysis results for dissolved metal cation concentrations after OER.

| Sample | Co (ppm) | Ni (ppm) | Fe (ppm) |
| --- | --- | --- | --- |
| CNF LDH | 0.082 | 0.061 | 0.173 |
| LDH\|\|VC\|\|Pc | 0.009 | 0.045 | 0.041 |
| LDH\|\|PCS\|\|Pc | 0.001 | 0.007 | 0.084 |

**Figure S31.** XRD patterns of a) CNF LDH, b) LDH||PCS||Pc, and c) LDH||VC||Pc before and after OER.


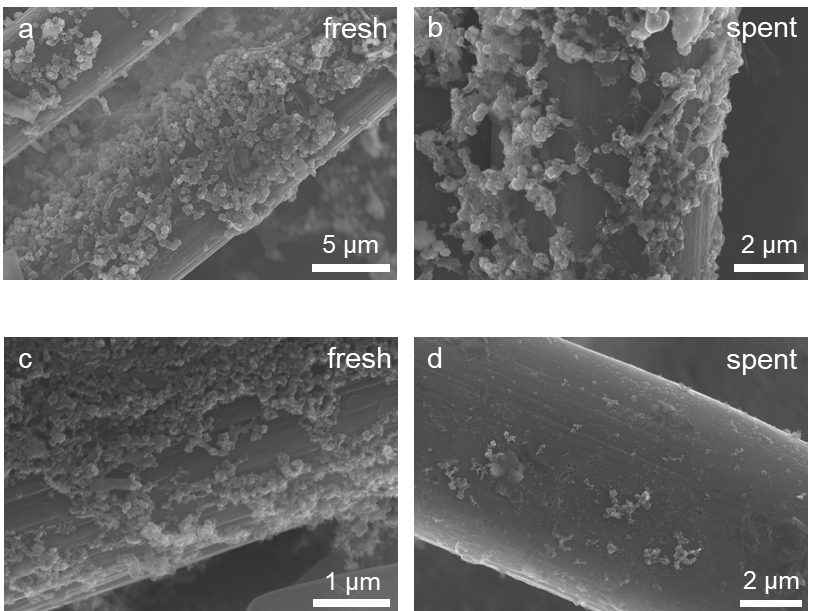


**Figure S32.** SEM images of a) fresh LDH||PCS||Pc, b) spent LDH||PCS||Pc, c) fresh LDH||VC||Pc, and d) spent LDH||VC||Pc.

Table S4. Performance comparison between LDH||PCS||Pc and other recently reported electrocatalysts.

| Catalyst | *E*_ORR_ at −3 mA cm^−2^  (V *vs.* RHE.) | *E*_OER_ at 10 mA cm^−2^  (V *vs.* RHE.) | Δ*E*  (V) | Ref. |
| --- | --- | --- | --- | --- |
| **LDH\|\|PCS\|\|Pc** | **0.88** | **1.51** | **0.63** | **This work** |
| Pt_1.1%_Fe_8.8%_Ni PF | 0.87 | 1.52 | 0.65 | [6] |
| Mo/C tubes | 0.81 | 1.55 | 0.74 | [7] |
| CoP | 0.86 | 1.56 | 0.7 | [8] |
| Cu-CoFS | 0.8 | 1.54 | 0.74 | [9] |
| FeCo SACs@Co/N-GC | 0.88 | 1.52 | 0.64 | [10] |
| CoNC SAC | 0.86 | 1.65 | 0.79 | [11] |
| V-Co_3_O_4_ | 0.82 | 1.58 | 0.76 | [12] |
| SA-PtCoF | 0.88 | 1.54 | 0.66 | [13] |
| Fe-N_4_ SAs/NPC | 0.88 | 1.66 | 0.78 | [14] |
| Pd/FeCo | 0.84 | 1.55 | 0.71 | [15] |
| NOGB-800 | 0.84 | 1.64 | 0.8 | [16] |
| FeCo-CNF | 0.87 | 1.63 | 0.76 | [17] |
| Co_9_S_8_@N,S-C | 0.88 | 1.53 | 0.65 | [18] |
| Co/Co2P@NCNTs | 0.9 | 1.58 | 0.68 | [19] |
| FeNi SAs/NC | 0.84 | 1.5 | 0.66 | [20] |
| Ni@N-HCGHF | 0.87 | 1.6 | 0.73 | [21] |
| NiSe_2_/CoSe_2_-N | 0.81 | 1.52 | 0.71 | [22] |
| Co/CNF (1000) | 0.89 | 1.55 | 0.66 | [23] |
| c-CoSe_2_-CoN/NC | 0.85 | 1.55 | 0.7 | [24] |
| FeCoNiMoW | 0.71 | 1.46 | 0.75 | [25] |
| pl-S-40 | 0.81 | 1.46 | 0.65 | [26] |
| NiFe-LDH/Fe1-N-C | 0.9 | 1.55 | 0.65 | [27] |
| CoS/Fe_3_S_4_@SNCP | 0.85 | 1.5 | 0.65 | [28] |
| Co/SP-NC | 0.86 | 1.57 | 0.71 | [29] |
| Co-CoN_4_@NCNs | 0.83 | 1.54 | 0.71 | [30] |
| CoN_4_-O/MX | 0.85 | 1.57 | 0.72 | [31] |
| O–Co–N/C | 0.85 | 1.52 | 0.67 | [32] |
| Fe SA/NCZ | 0.87 | 1.55 | 0.68 | [33] |
| Fe@CNx | 0.84 | 1.64 | 0.8 | [34] |
| SA&NP-FeCo-NTS | 0.87 | 1.58 | 0.71 | [35] |
| Co_2_-N-HCS-900 | 0.86 | 1.56 | 0.7 | [36] |
| Co-N-CTS | 0.86 | 1.51 | 0.65 | [37] |
| Co-CNHSC-3 | 0.84 | 1.58 | 0.74 | [38] |
| Co-HTA-CN | 0.8 | 1.58 | 0.78 | [39] |


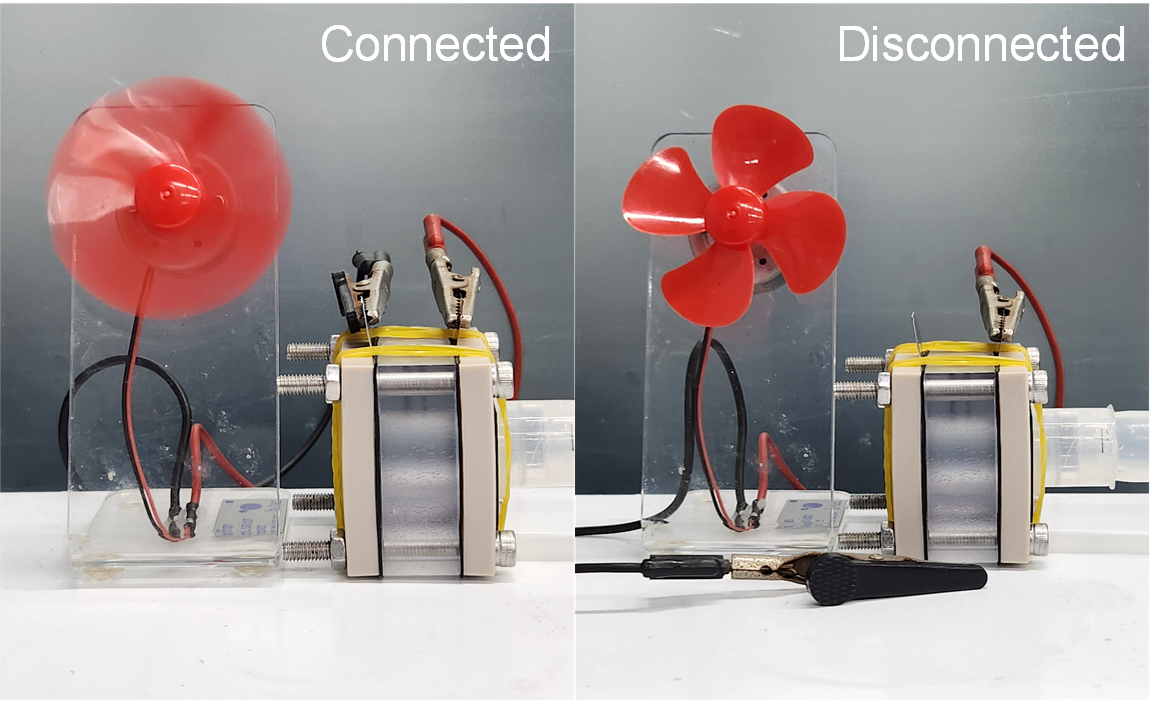


Figure S33. Photograph of the fan powered by the LDH||PCS||Pc-based home-assembled ZAB cell.

Figure S34. Polarization discharge-charge curves and power density for LDH||PCS||Pc- and IrO_2_+Pt/C-based ZAB cells.


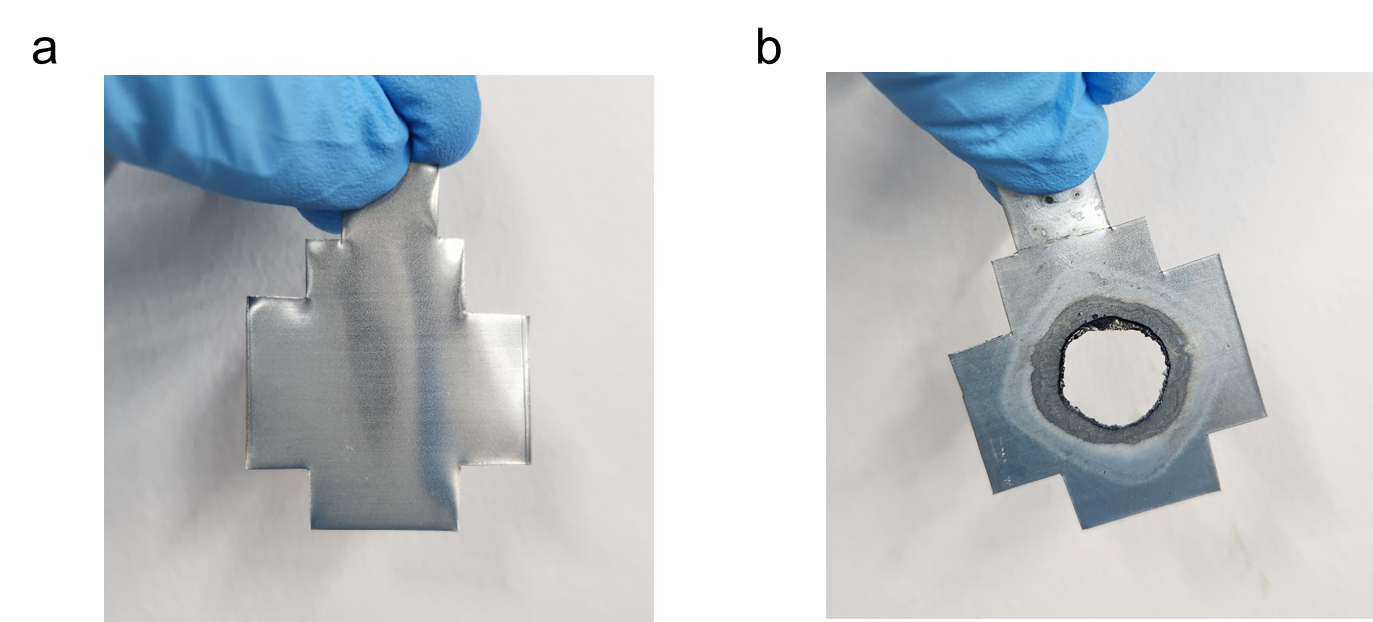


Figure S35. Photographs of the a) initial and b) spent zinc electrode for measuring specific capacity of zinc-air battery.

Figure S36. Rate capability evaluating of LDH||PCS||Pc- and IrO_2_+Pt/C-based ZABs at different rates from 5 to 30 mA cm^−2^.

Figure S37. Discharge-charge curves with 6 min cycles at a current density of 5 mA cm^−2^ for LDH||PCS||Pc- and IrO_2_+Pt/C-based ZAB cells.


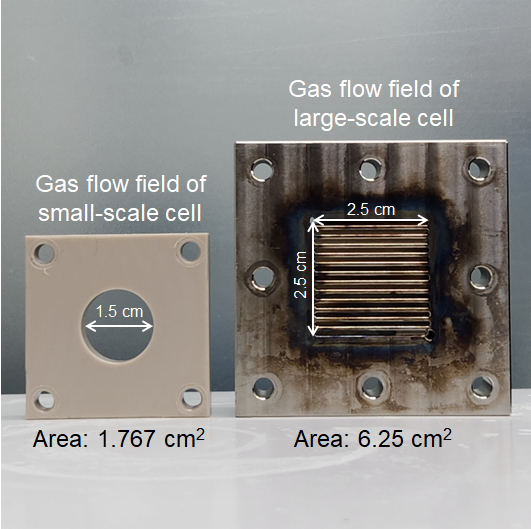


Figure S38. Photographs of the gas flow field of small-scale and large-scale ZAB cells


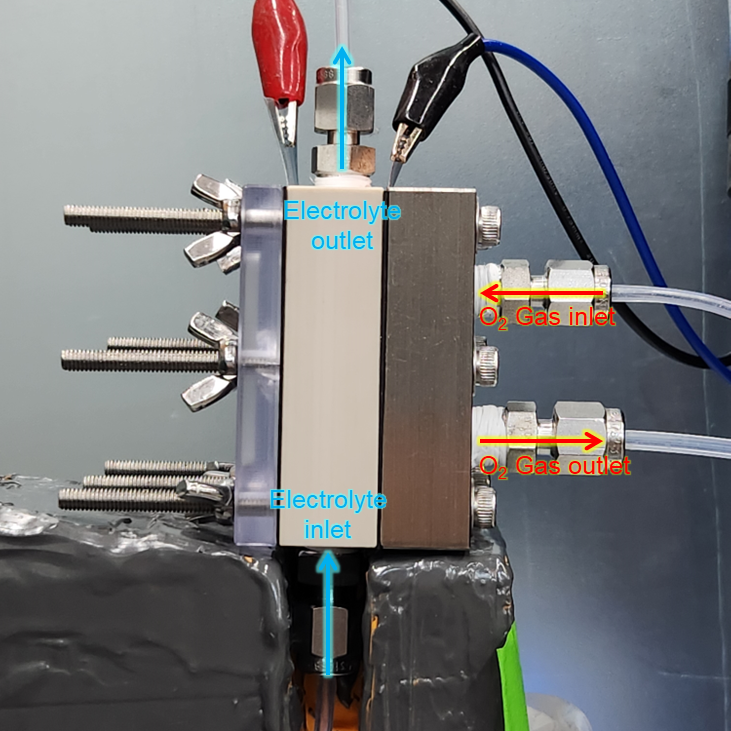


Figure S39. Photographs of the large-scale cell showing gas and electrolyte flowing direction.

**Figure S40.** a) LSV curves for determining a) OER and b) ORR electrochemical activity of LDH||PCS||Pc nanocomposite and IrO_2_+Pt/C mixture as reference catalyst. c) Electron transfer numbers determined by RRDE method for LDH||PCS||Pc and IrO_2_+Pt/C mixture (1:1 mass ratio).

**Figure S41.** a) Polarization discharge-charge curves and power density for LDH||PCS||Pc- and IrO_2_+Pt/C-based large-scale ZAB cells. b) Comparison of the discharge curves and power density of LDH||PCS||Pc-based large-scale ZAB cells based on measurements of three different devices under identical condition. c) Comparison of the discharge curves of LDH||PCS||Pc-based large-scale ZAB cells between O_2_ and Ar gaseous reactant environments.


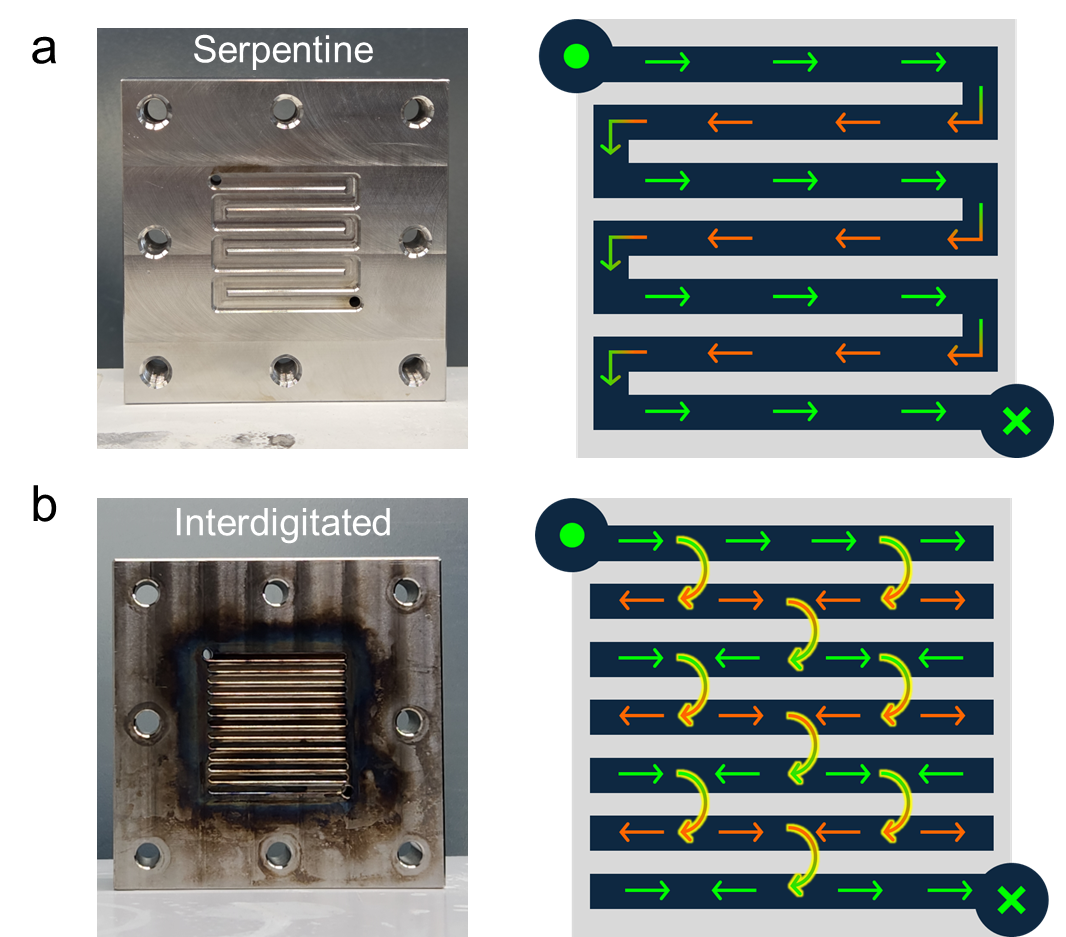


**Figure S42.** Photographic images and schematic illustrations of (a) serpentine and (b) interdigitated gas flow fields. In the schematic illustrations, the circular and cross symbols represent the gas inlet and outlet, respectively.

**Figure S43.** Comparison of a) discharge and power density curves, and b) peak power density of LDH||PCS||Pc-based large-scale ZAB cells under 300 mL min^−1^ and 100 mL min^−1^ of O_2_ gas flow rate.

**Figure S44.** Specific capacity and maximum capacity for LDH||PCS||Pc-based large-scale ZAB cells.


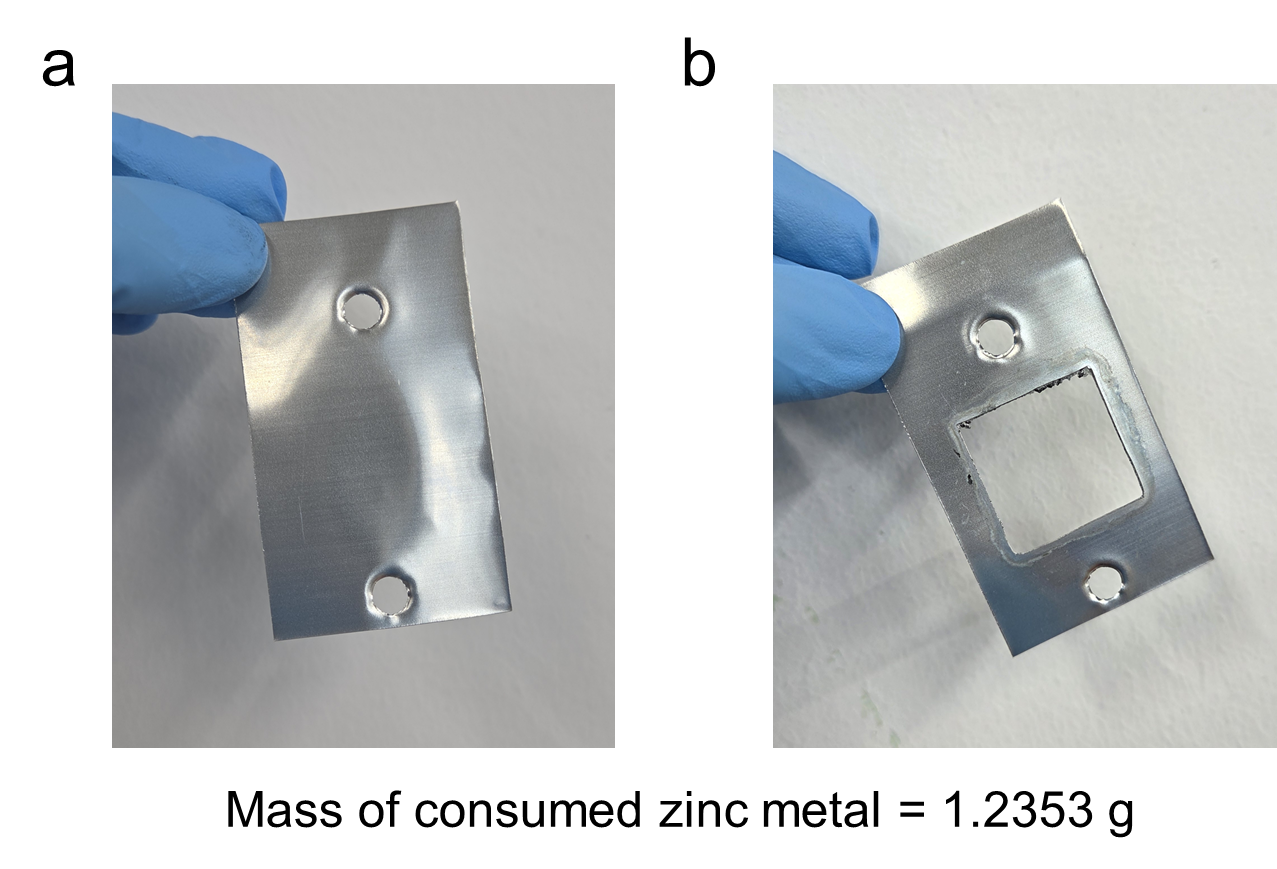


**Figure S45.** Photographs of the a) initial and b) spent zinc electrode for measuring specific capacity of LDH||PCS||Pc-based large-scale ZAB.

Figure S46. a) Galvanostatic discharge-charge curves with 10 min cycles at a current density of 5 mA cm^−2^ of large-scale LDH||PCS||Pc- and IrO_2_+Pt/C-based ZABs. b) Enlarged galvanostatic discharge-charge curves of large-scale LDH||PCS||Pc-based ZAB at the 0, and 300 h.

**Figure S47.** Galvanostatic discharge-charge curves at current densities of 5, 10, 20, and 5 mA cm^−2^, each for 25 h, for LDH||PCS||Pc-based large-scale ZAB cells.

Figure S48. Back-ground corrected chronoamperometry curve of the RRDE at −0.3 V *vs.* Ag/AgCl with an electrode rotation speed of 1600 rpm in Ar-saturated 0.1 м KOH + 2 mм K_3_[Fe(CN)_6_] electrolyte.

**References**

[1] a) K. S. Park, Z. Ni, A. P. Côté, J. Y. Choi, R. Huang, F. J. Uribe-Romo, H. K. Chae, M. O’Keeffe, O. M. Yaghi, *Proc. Natl. Acad. Sci.* **2006**, *103* (27), 10186; b) R. Banerjee, A. Phan, B. Wang, C. Knobler, H. Furukawa, M. O'Keeffe, O. M. Yaghi, *Science* **2008**, *319* (5865), 939.

[2] J. Yang, F. Zhang, H. Lu, X. Hong, H. Jiang, Y. Wu, Y. Li, *Angew. Chem., Int. Ed.* **2015**, *127* (37), 11039.

[3] B. Liu, H. Shioyama, T. Akita, Q. Xu, *J. Am. Chem. Soc.* **2008**, *130* (16), 5390.

[4] K. R. Paton, E. Varrla, C. Backes, R. J. Smith, U. Khan, A. O’Neill, C. Boland, M. Lotya, O. M. Istrate, P. King, T. Higgins, S. Barwich, P. May, P. Puczkarski, I. Ahmed, M. Moebius, H. Pettersson, E. Long, J. Coelho, S. E. O’Brien, E. K. McGuire, B. M. Sanchez, G. S. Duesberg, N. McEvoy, T. J. Pennycook, C. Downing, A. Crossley, V. Nicolosi, J. N. Coleman, *Nat. Mater.* **2014**, *13* (6), 624.

[5] M. Yi, Z. Shen, *Carbon* **2014**, *78*, 622.

[6] G. Wang, J. Chang, S. Koul, A. Kushima, Y. Yang, *J. Am. Chem. Soc.* **2021**, *143* (30), 11595.

[7] Y. Zhao, Z. Zhang, L. Liu, Y. Wang, T. Wu, W. Qin, S. Liu, B. Jia, H. Wu, D. Zhang, X. Qu, G. Qi, E. P. Giannelis, M. Qin, S. Guo, *J. Am. Chem. Soc.* **2022**, *144* (45), 20571.

[8] H. Li, Q. Li, P. Wen, T. B. Williams, S. Adhikari, C. Dun, C. Lu, D. Itanze, L. Jiang, D. L. Carroll, G. L. Donati, P. M. Lundin, Y. Qiu, S. M. Geyer, *Adv. Mater.* **2018**, *30* (9), 1705796.

[9] Z. Li, Q. Wang, X. Bai, M. Wang, Z. Yang, Y. Du, G. E. Sterbinsky, D. Wu, Z. Yang, H. Tian, F. Pan, M. Gu, Y. Liu, Z. Feng, Y. Yang, *Energy Environ. Sci.* **2021**, *14* (9), 5035.

[10] N. K. Wagh, D.-H. Kim, S.-H. Kim, S. S. Shinde, J.-H. Lee, *ACS Nano* **2021**, *15* (9), 14683.

[11] C.-X. Zhao, J.-N. Liu, J. Wang, C. Wang, X. Guo, X.-Y. Li, X. Chen, L. Song, B.-Q. Li, Q. Zhang, *Sci. Adv.* **2022**, *8* (11), eabn5091.

[12] Y. Rao, S. Chen, Q. Yue, Y. Kang, *ACS Catal.* **2021**, *11* (13), 8097.

[13] J. Yan, Y. Wang, Y. Zhang, S. Xia, J. Yu, B. Ding, *Adv. Mater.* **2021**, *33* (5), 2007525.

[14] Y. Pan, S. Liu, K. Sun, X. Chen, B. Wang, K. Wu, X. Cao, W. Cheong, R. Shen, A. Han, Z. Chen, L. Zheng, J. Luo, Y. Lin, Y. Liu, D. Wang, Q. Peng, Q. Zhang, C. Chen, Y. Li, *Angew. Chem., Int. Ed.* **2018**, *57* (28), 8614.

[15] F. Pan, Z. Li, Z. Yang, Q. Ma, M. Wang, H. Wang, M. Olszta, G. Wang, Z. Feng, Y. Du, Y. Yang, *Adv. Energy Mater.* **2021**, *11* (3), 2002204.

[16] Q. Hu, G. Li, G. Li, X. Liu, B. Zhu, X. Chai, Q. Zhang, J. Liu, C. He, *Adv. Energy Mater.* **2019**, *9* (14), 1803867.

[17] Y. Wang, Z. Li, P. Zhang, Y. Pan, Y. Zhang, Q. Cai, S. R. P. Silva, J. Liu, G. Zhang, X. Sun, Z. Yan, *Nano energy* **2021**, *87*, 106147.

[18] D. Lyu, S. Yao, A. Ali, Z. Q. Tian, P. Tsiakaras, P. K. Shen, *Adv. Energy Mater.* **2021**, *11* (28), 2101249.

[19] M. Wu, G. Zhang, N. Chen, Y. Hu, T. Regier, D. Rawach, S. Sun, *ACS Energy Lett.* **2021**, *6* (4), 1153.

[20] D. Yu, Y. Ma, F. Hu, C. C. Lin, L. Li, H. Y. Chen, X. Han, S. Peng, *Adv. Energy Mater.* **2021**, *11* (30), 2101242.

[21] L. Yan, Y. Xu, P. Chen, S. Zhang, H. Jiang, L. Yang, Y. Wang, L. Zhang, J. Shen, X. Zhao, L. Wang, *Adv. Mater.* **2020**, *32* (48), 2003313.

[22] X. Zheng, X. Han, Y. Cao, Y. Zhang, D. Nordlund, J. Wang, S. Chou, H. Liu, L. Li, C. Zhong, Y. Deng, W. Hu, *Adv. Mater.* **2020**, *32* (26), 2000607.

[23] Z. Yang, C. Zhao, Y. Qu, H. Zhou, F. Zhou, J. Wang, Y. Wu, Y. Li, *Adv. Mater.* **2019**, *31* (12), 1808043.

[24] X. Xu, X. Wang, S. Huo, X. Liu, X. Ma, M. Liu, J. Zou, *Adv. Mater.* **2024**, *36* (8), 2306844.

[25] R. He, L. Yang, Y. Zhang, D. Jiang, S. Lee, S. Horta, Z. Liang, X. Lu, A. O. Moghaddam, J. Li, M. Ibáñez, Y. Xu, Y. Zhou, A. Cabot, *Adv. Mater.* **2023**, *35* (46), 2303719.

[26] Z. Shao, Q. Zhu, Y. Sun, Y. Zhang, Y. Jiang, S. Deng, W. Zhang, K. Huang, S. Feng, *Adv. Mater.* **2022**, *34* (15), 2110172.

[27] Z. Q. Liu, X. Liang, F. X. Ma, Y. X. Xiong, G. Zhang, G. Chen, L. Zhen, C. Y. Xu, *Adv. Energy Mater.* **2023**, *13* (13), 2203609.

[28] L. Yan, B. Xie, C. Yang, Y. Wang, J. Ning, Y. Zhong, Y. Hu, *Adv. Energy Mater.* **2023**, *13* (10), 2204245.

[29] H. Chang, X. Liu, S. Zhao, Z. Liu, R. Lv, Q. Zhang, T. F. Yi, *Adv. Funct. Mater.* **2024**, *34* (16), 2313491.

[30] K. Ding, J. Hu, J. Luo, L. Zhao, W. Jin, Y. Liu, Z. Wu, G. Zou, H. Hou, X. Ji, *Adv. Funct. Mater.* **2022**, *32* (52), 2207331.

[31] Y. L. Zhang, B. Liu, Y. K. Dai, Y. F. Xia, P. Guo, Y. Y. Liu, F. Kong, Q. Zhang, L. Zhao, Z. B. Wang, *Adv. Funct. Mater.* **2022**, *32* (49), 2209499.

[32] W. Zhang, C. H. Xu, H. Zheng, R. Li, K. Zhou, *Adv. Funct. Mater.* **2022**, *32* (23), 2200763.

[33] C. Jiao, Z. Xu, J. Shao, Y. Xia, J. Tseng, G. Ren, N. Zhang, P. Liu, C. Liu, G. Li, *Adv. Funct. Mater.* **2023**, *33* (20), 2213897.

[34] J. Quílez‐Bermejo, S. García‐Dalí, A. Daouli, A. Zitolo, R. L. Canevesi, M. Emo, M. T. Izquierdo, M. Badawi, A. Celzard, V. Fierro, *Adv. Funct. Mater.* **2023**, *33* (21), 2300405.

[35] Q. Zhang, P. Liu, X. Fu, Y. Yuan, L. Wang, R. Gao, L. Zheng, L. Yang, Z. Bai, *Adv. Funct. Mater.* **2022**, *32* (22), 2112805.

[36] X. Wang, L. Xu, C. Li, C. Zhang, H. Yao, R. Xu, P. Cui, X. Zheng, M. Gu, J. Lee, *Nat. Commun.* **2023**, *14* (1), 7210.

[37] W. Shi, Z. Li, Z. Gong, Z. Liang, H. Liu, Y.-C. Han, H. Niu, B. Song, X. Chi, J. Zhou, H. Wang, B. Y. Xia, Y. Yao, Z.-Q. Tian, *Nat. Commun.* **2023**, *14* (1), 2294.

[38] W. Zhang, X. Guo, C. Li, J. Y. Xue, W. Y. Xu, Z. Niu, H. Gu, C. Redshaw, J. P. Lang, *Carbon Energy* **2023**, *5* (8), e317.

[39] S. Liu, M. Liu, X. Li, S. Yang, Q. Miao, Q. Xu, G. Zeng, *Carbon Energy* **2023**, *5* (5), e303.
